# Design of D-Amino Acids SARS-CoV-2 Main Protease Inhibitors Using the Cationic Peptide from Rattlesnake Venom as a Scaffold

**DOI:** 10.3390/ph15050540

**Published:** 2022-04-27

**Authors:** Raphael J. Eberle, Ian Gering, Markus Tusche, Philipp N. Ostermann, Lisa Müller, Ortwin Adams, Heiner Schaal, Danilo S. Olivier, Marcos S. Amaral, Raghuvir K. Arni, Dieter Willbold, Mônika A. Coronado

**Affiliations:** 1 Institute of Biological Information Processing (IBI-7: Structural Biochemistry), Forschungszentrum Jülich, 52428 Jülich, Germany; r.eberle@fz-juelich.de (R.J.E.); i.gering@fz-juelich.de (I.G.); m.tusche@fz-juelich.de (M.T.); d.willbold@fz-juelich.de (D.W.); 2 Institut für Physikalische Biologie, Heinrich-Heine-Universität Düsseldorf, Universitätsstraße, 40225 Düsseldorf, Germany; 3 Institute of Virology, Medical Faculty, University Hospital Düsseldorf, Heinrich-Heine-Universität Düsseldorf, 40225 Düsseldorf, Germany; philipp.ostermann@uni-duesseldorf.de (P.N.O.); lisa.mueller2@med.uni-duesseldorf.de (L.M.); ortwin.adams@uni-duesseldorf.de (O.A.); schaal@uni-duesseldorf.de (H.S.); 4 Integrated Sciences Center, Campus Cimba, Federal University of Tocantins, Araguaína 77824-838, TO, Brazil; d.olivier@gmail.com; 5 Institute of Physics, Federal University of Mato Grosso do Sul, Campo Grande 79070-900, MS, Brazil; marcos.amaral@ufms.br; 6 Multiuser Center for Biomolecular Innovation, Department of Physics, IBILCE, Universidade Estadual Paulista (UNESP), São José do Rio Preto 15054-000, SP, Brazil; arni@sjrp.unesp.br; 7 JuStruct: Jülich Centre for Structural Biology, Forschungszentrum Jülich, 52428 Jülich, Germany

**Keywords:** SARS-CoV-2, COVID-19, 3CL^pro^, main protease, inhibitor, crotamine derivative peptides, D-peptides

## Abstract

The C30 endopeptidase (3C-like protease; 3CL^pro^) is essential for the life cycle of SARS-CoV-2 (severe acute respiratory syndrome-coronavirus-2) since it plays a pivotal role in viral replication and transcription and, hence, is a promising drug target. Molecules isolated from animals, insects, plants, or microorganisms can serve as a scaffold for the design of novel biopharmaceutical products. Crotamine, a small cationic peptide from the venom of the rattlesnake *Crotalus durissus terrificus*, has been the focus of many studies since it exhibits activities such as analgesic, in vitro antibacterial, and hemolytic activities. The crotamine derivative L-peptides (L-CDP) that inhibit the 3CL protease in the low µM range were examined since they are susceptible to proteolytic degradation; we explored the utility of their D-enantiomers form. Comparative uptake inhibition analysis showed D-CDP as a promising prototype for a D-peptide-based drug. We also found that the D-peptides can impair SARS-CoV-2 replication in vivo, probably targeting the viral protease 3CL^pro^.

## 1. Introduction

In Wuhan, Hubei Province, China, in December 2019, a rapid increase in the number of pneumonia suspect cases [1] quickly aroused interest, and it sounded the emergency call to the World Health Organization (WHO) in January 2020 as a “public health emergency of international concern”. The resulting disease, Coronavirus Disease-2019 (COVID-19), exploded into a global pandemic within a few months, claiming lives on all continents. SARS-Coronavirus-2 (SARS-CoV-2) has resulted in over 430 million confirmed cases and over 5.9 million deaths worldwide, as reported by the WHO on 25 February 2022 [2]. The worldwide vaccination campaign uses clinically safe and efficient vaccines against SARS-CoV-2 (e.g., BioNTech-Pfizer, Moderna, Johnson & Johnson, and AstraZeneca vaccines) [3,4,5,6], and so far, more than 10 billion vaccine doses have been administered [2].

Several candidate drugs that may inhibit SARS-CoV-2 infection and replication have been approved for emergency use (e.g., Remdesivir, Dexamethasone, Favipiravir, Lopinavir/Ritonavir, and Darunavir) [7,8,9,10,11]. Given the considerable limitation of direct-acting antivirals for COVID-19 and an increasing presence of SARS-CoV-2 variants (B.1.617.2, B.1.1.7, B.1.351, A.23.1, B.1.525, B.1.526 and P.1) [12], it remains a strategic priority to develop new candidates with minimal side effects, which are also targeted against new variants. Upon entering and uncoating the viral particles, the positive-stranded RNA genome is rapidly translated into two polyproteins processed (pp1a and pp1ab) by 3CL and papain-like proteases into 16 nonstructural proteins (NSPs) [13,14]. 3CL^pro^ is a cysteine protease organised in three domains (domains I to III) with a chymotrypsin-like fold [15]. Its active form consists of two protomers (homodimer) containing a noncanonical Cys-His dyad located in the cleft between domains I and II [15,16,17]. The functional importance of 3CL^pro^ in the viral life cycle, combined with the absence of closely related homologues in humans, indicates that this protease is an attractive target for developing antiviral drugs [18].

In recent years, antimicrobial peptides (AMPs) have been considered to hold promise as a viable solution to form the basis for the design of novel peptides to combat hazardous microorganism infections. The use of AMPs can be promising as a therapeutic tool to address increasing viral infections, for which no current or authorised medication or treatment is available [19]. AMPs are frequently used to treat viral-related diseases such as Zika (ZIKV), Dengue (DENV) [20], and Influenza A virus infection (IAV) [21].

Crotamine (Cro), a small cationic polypeptide originally encountered in the venom of the South American rattlesnake, *Crotalus durissus terrificus* [22,23], possesses cell wall penetrating properties [24,25], and several biological functions of this polypeptide were described, including antimicrobial, antifungal, and antitumoral activities [24,25,26,27]. These properties were mainly determined by the overall positive net surface charge distribution of crotamine [24,25,26,27]. Characterised as a novel cell-penetrating polypeptide (CPP) nanocarrier, Cro has biotechnological applications due to its peculiar specificity for highly proliferating cells. [24,25,28]. Like other CPPs, Cro showed a rapid translocation efficiency (within 5 min) into all cell types investigated [29]. A small peptide composed of 42 amino acid residues (YKQCHKKGGHCFPKEKICLPPSSDFGKMDCRWRWKCCKKGSG) [22,23] containing two putative nuclear localization sequence (NLS) motifs Cro_2–18 (KQCHKKGGHCFPKEKIC) and Cro_27–39 (KMDCRWRWKCCKK) [29], the sequence Crot_27–39 was selected and named L-CDP1 as the initial sequence for inhibition studies against SARS-CoV-2 3CL^pro^. Based on the first sequence, several crotamine derivative peptides (CDPs) in L-form were specifically modified, and D-forms were designed. They were tested concerning their inhibitory potential against the virus’ main protease.

## 2. Results and Discussion

SARS-CoV-2, the causative agent of COVID-19, is still a significant threat to public health globally because of the high infection rates. In this study, we investigated the mechanism of a peptide and its D-enantiomer against the main protease and their intracellular antiviral activity against SARS-CoV-2.

### 2.1. Preparation of SARS-CoV-2 3CL^pro^

The SARS-CoV-2 3CL^pro^_GST fusion protein was expressed in *E. coli* Lemo21 (DE3) cells and purified using a GSH-Sepharose column (Supplementary Figure S1A). The relevant protein fractions were concentrated and prepared for PreScission protease cleavage to remove the GST-tag. The SDS gel (Supplementary Figure S1) indicates the cleavage efficiency and the purity of 3CL^pro^.

### 2.2. Primary Inhibition Assay of Crotamine and L-CDPs against SARS-CoV-2 3CL^pro^

In order to identify a novel inhibitor of 3CL^pro^, we used as a scaffold two sequences of the small cationic polypeptide (crotamine) for initial inhibition studies.

The L-CDP1 (wild type sequence Cro_27–39) includes three cysteine residues. The L-CDP2-9 are modified peptides by substituting the cysteine to serine residues in a different position (Table 1 and Supplementary Figures S2 and S3). As the cysteine residues do not tolerate substitution by other amino acids, we used the tolerability to replace just the thiol group, as cysteine differs from serine in a single atom. In principle, the positively charged residues were maintained, as it gives the cell-penetrating characteristic of crotamine. Positive charge residue is postulated to facilitate the penetration into cells across the cell membrane due to their membrane modification properties [30].

**Figure 1:**
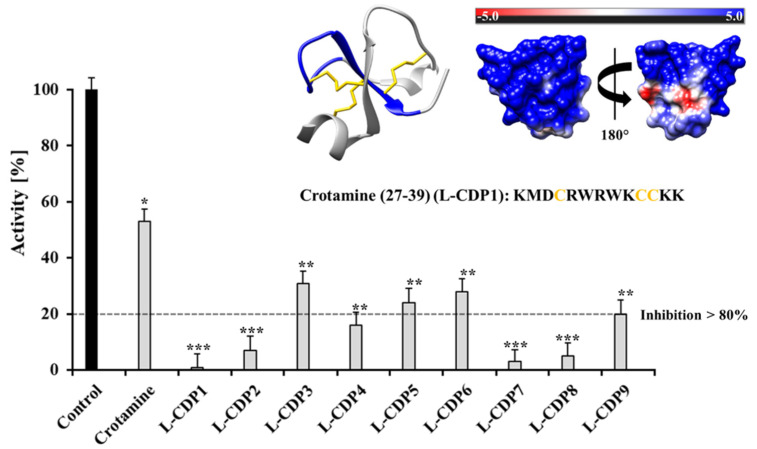
Primary inhibition tests of crotamine and L-CDPs against SARS-CoV-2 3CL^pro^. Crotamine inhibits the virus protease activity by around 50%. L-CDP1, L-CDP2, L-CDP4, L-CDP7, and L-CDP8 inhibit the virus protease activity by more than 80%. Data shown are the mean ± SD from 3 independent measurements (*n* = 3). Asterisks mean that the data differs from the control (0 µM inhibitor) significantly at *p* < 0.05 (*), *p* < 0.01 (**), and *p* < 0.001 (***) level according to ANOVA and Tukey’s test. The experimental model of crotamine is shown in coulombic surfaces and cartoons, with the L-CDP1 sequence highlighted in blue (PDB entry: 4GV5).

The SARS-CoV-2 3CL^pro^ activity assay was performed using DABCYL-KTSAVLQ/SGFRKME-EDANS (Bachem, Switzerland) as substrate [31,32,33,34]. A primary inhibition test with Cro and the L-CDPs (30 µM) was performed to screen the best inhibitor peptide against the virus protease (Figure 1). An additional experiment was performed where the substrate and the inhibitor were added simultaneously (without incubation), but no significant differences in the results could be observed.

The primary inhibition tests revealed a strong effect of L-CDP1, L-CDP2, L-CDP7, and L-CDP8 peptides against the SARS-CoV-2 3CL^pro^ activity assay, and those peptides inhibit the virus protease activity by more than 80%. All peptides were subject to the dose-dependent studies (Supplementary Figure S4); however, the peptides that present more than 80% inhibition have been chosen for further studies.

### 2.3. Characterisation of the 3CL^pro^ Inhibition by Crotamine and L-CDPs

**Figure 2:**
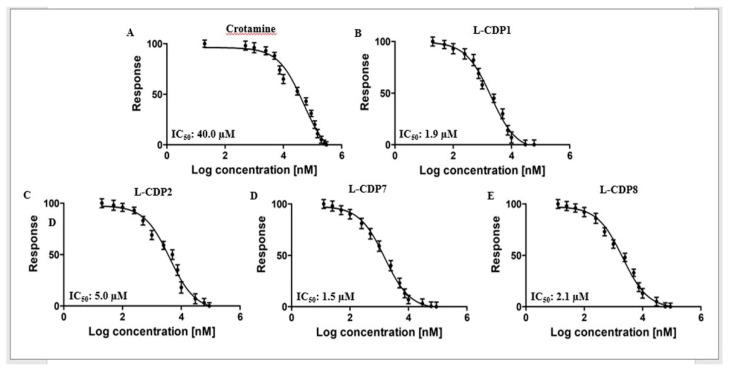
Crotamine, L-CDP1, L-CDP2, L-CDP7, and L-CDP8 inhibitory activity against SARS-CoV-2 3CL^pro^. Dose-response curves for IC_50_ determination. The normalised response [%] of SARS-CoV-2 3CL^pro^ is plotted against the log of the inhibitor concentration. (**A**): Dose-response curve of crotamine and SARS-CoV-2 3CL^pro^. (**B**): Dose-response curve of L-CDP1 and SARS-CoV-2 3CL^pro^. (**C**): Dose-response curve of L-CDP2 and SARS-CoV-2 3CL^pro^. (**D**): Dose-response curve of L-CDP7 and SARS-CoV-2 3CL^pro^. (**E**): Dose-response curve of L-CDP8 and SARS-CoV-2 3CL^pro^. Data shown are the mean ± SD from three independent measurements (*n* = 3).

The selected peptides were further analysed with respect to their actual potential to inhibit the catalytic activity of 3CL^pro^ in a biochemical assay. To gain insight into functional implications caused by the selected peptide (L-CDP1), we checked the minimum concentration of Cro required to inhibit 100% of the protease activity. The full-length Cro was tested using a concentration range of 0–300 µM, and the polypeptide demonstrated 100% protease inhibition at a concentration of 300 µM (Supplementary Figure S4A), presenting an IC_50_ value of 40 ± 3.1 µM (Table 1, Figure 2A). In contrast, the selected wild-type sequence Crot_27–39 (L-CDP1) inhibits 100% of the recombinant SARS-CoV-2 protease activity at a concentration of 30 µM (Supplementary Figure S4B) with an IC_50_ value of 1.9 ± 0.3 µM (Table 1, Figure 2B). The replacement of all cysteine residues in L-CDP1 by serine residues, a peptide named L-CDP2 (Table 1), led to an increase in IC_50_ values (5.0 ± 0.8 µM), revealing that the cysteine residue can increase the inhibition rate of the protease (Figure 2C and Supplementary Figure S4C). The crotamine derivative L-peptide-7, a peptide that presents an amino acid substitution, Cys36 (WT numbering) to Ser, and L-CDP8 (Cys37 to Ser) (see Table 1), inhibits the protease by 100% at 60 µM concentration (Supplementary Figure S4H,I) with IC_50_ values of 1.5 ± 0.4 and 2.1 ± 0.4 µM (Table 1, Figure 2D,E), respectively. Supplementary Figure S4 shows the inhibition effect for all modified L-CDP against the 3CL protease. Supplementary Figure S5 shows the dose-response curves for IC_50_ determination for the remaining modified peptides (L-CDP3, 4, 5, 6, and 9).

Regarding residues substitution (Table 1), the secondary structure of L-CDP1, L-CDP2, L-CDP7, and L-CDP8 was investigated by circular dichroism (CD), demonstrating that the substitution of the cysteine residue will result in conformational changes (Supplementary Figure S6). As expected, a difference in uptake behaviour was observed with only one cysteine residue replacement, as observed by IC_50_ values (Table 1 and Figure 2C). However, the substitution at position 36 (WT numbering) increases the inhibitory efficiency of the peptide L-CDP7 (Table 1 and Figure 2D). Table 1 summarises the experiments performed with the modified L-CDP peptides, suggesting that these features are crucial for high affinity.

**Figure 3:**
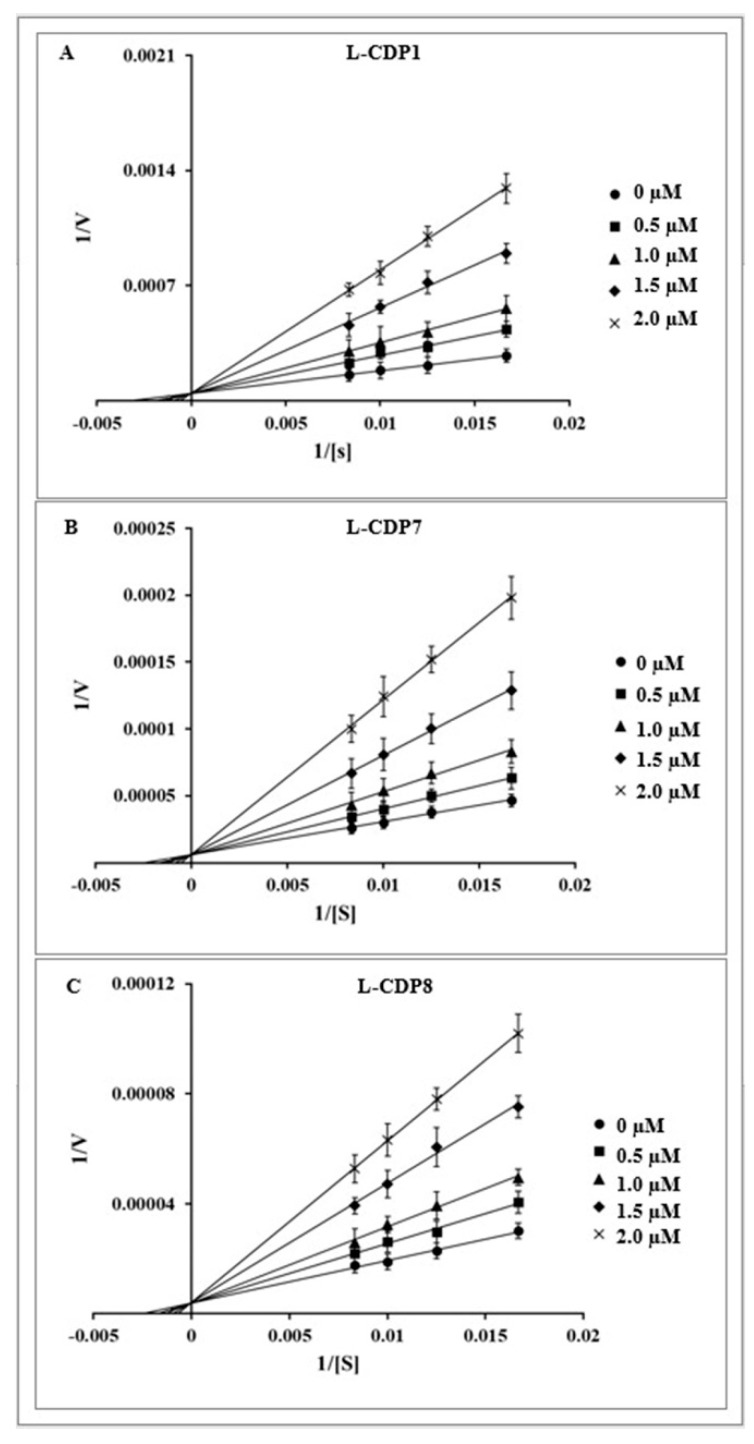
Inhibition mode of L-CDP1, L-CDP7, and L-CDP8 over SARS-CoV-2 3CL^pro^. Lineweaver-Burk plots to determine the inhibition modes are presented. [S] is the substrate concentration; v is the initial reaction rate. (**A**): Lineweaver-Burk plot for L-CDP1 inhibition of SARS-CoV-2 3CL^pro^. (**B**): Lineweaver-Burk plot for L-CDP7 inhibition of SARS-CoV-2 3CL^pro^. (**C**): Lineweaver-Burk plot for L-CDP8 inhibition of SARS-CoV-2 3CL^pro^. Data shown are the mean ± SD from 3 independent measurements (*n* = 3).

Based on the IC_50_ values, we proceeded only with the peptides that presented IC_50_ close to 2 µM. The selected peptides (L-CDP1, 7 and 8) were then analysed for their mode of inhibition (Table 1). Detailed mechanistic studies using a fluorescence-based protease assay were performed, indicating that L-CDP1, L-CDP7, and L-CDP8 peptides are competitive inhibitors (Figure 3). The results indicate that these peptides interact directly with amino acid residues located in the active site or with amino acids located in the substrate-binding region of the protease, consequently precluding substrate entry to the active site. Since L-CDP1 and L-CDP7 present IC_50_ values < 2 µM, they were considered for profound evaluation.

### 2.4. The Binding Affinity of the L-CDP1 and L-CDP7 Using Surface Plasmon Resonance

We further investigated the binding affinity of L-CDP1 and L-CDP7 molecules using Surface Plasmon Resonance (SPR) (Table 2, Supplementary Figure S7). The protease was passed over a CM-5 sensor chip pre-immobilised with the individual L-peptides (L-CDP1 and L-CDP7). L-CDP1 had an equilibrium rate constant (K_D_) of 65 ± 20.1 nM, whereas L-CDP7 shows 304 ± 70.3 nM, also illustrated in Table 2. From the K_D_ value obtained from SPR, it was observed that 3CL^pro^ had a higher binding efficacy for the L-CDP1 receptor.

The observed changes in the dissociation process can be attributed to a distinct aspect that the substitution of a sulfur-containing amino acid (Cysteine) by a hydroxyl group (Serine) changes the secondary structure (as described before), as well as the mode of interaction of the peptide with the target protease. Jha et al. described in an internalisation study using a Cro derivative peptide (CyLoP-1) that the substitution or deletion of the cysteine residue reduces cellular uptake and cytosolic distribution [35]. Both studies show the importance of the cysteine residue, not only for internalisation but also for inhibiting the SARS-CoV-2 protease.

### 2.5. Characterization of the CDP1 and CDP7 D-Peptide Enantiomers

**Figure 4:**
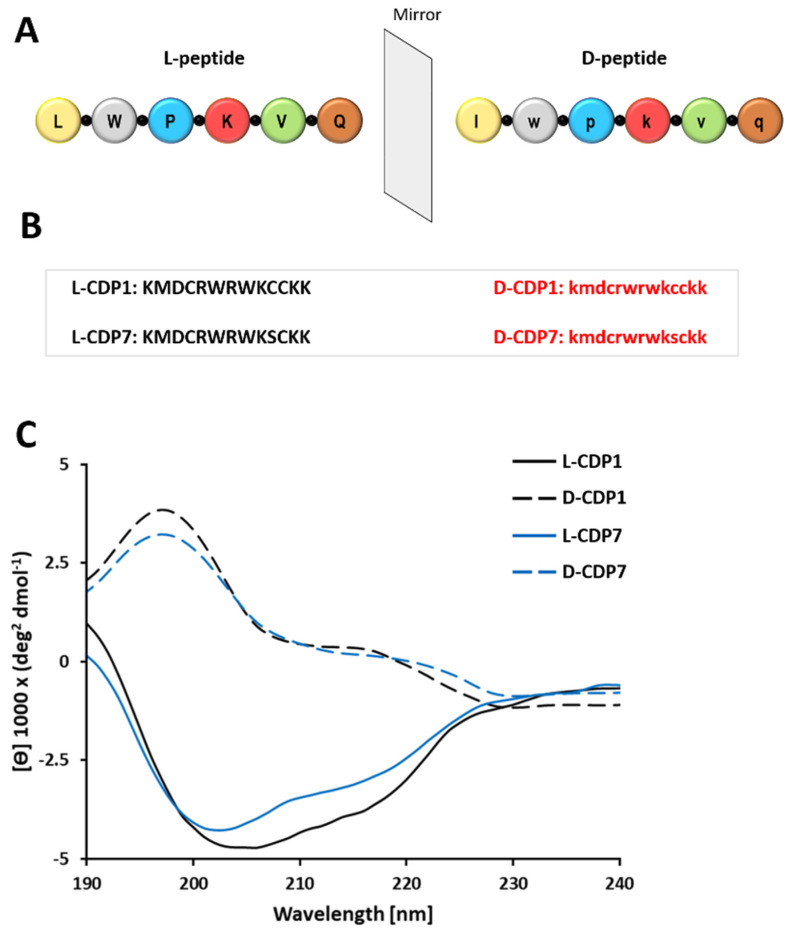
Principle of D-peptides. (**A**): Schematic diagram of D-peptides. (**B**): Sequences of D-CDP1, D-CDP7, and their mother L-peptides. (**C**): Circular dichroism (CD) spectroscopy of L- and D-CDP peptides. The CD spectrum of D-peptide in solution is presented as molar ellipticity [θ].

As attractive drug candidates, peptides have increasingly become leading molecules in drug development. However, their application is limited due to the susceptibility of L-peptides to endogenous enzymes. On the other hand, peptides composed of D-amino acids rarely undergo the process of proteolysis. In order to conserve all the essential biological properties of the L-enantiomers peptides against human protease degradations, CDP1 and CDP7 were synthesised in D-enantiomers formed by creating a mirror image of the mother L-peptides (Figure 4A,B). D-peptides, when compared to their L-enantiomeric equivalents, possess several therapeutic advantages. As shown previously, the proteolytic stability of D-peptides is superior to L-peptides, which can dramatically increase serum half-life [36,37], resulting in reduced immunogenicity and increased bioavailability of the D-peptides [38]. Welch et al. 2007 described promising D-peptide inhibitors that effectively inhibit human immunodeficiency virus 1 (HIV-1) entry [39].

L-CDP peptides are susceptible to hydrolysis by proteases, which restricts their therapeutic utility. CDP1 and CDP7 were synthesised (Genscript) in D-enantiomeric form seeking stability based on the mirror symmetry. We performed circular dichroism (CD) measurements of both D-CDP1 and D-CDP7 in solution to confirm that the D-peptides were in the correct enantiomeric form. As expected, the CD spectra of the D-peptides is the mirror image of that of L-peptides (Figure 4C).

As protein hydrolysis is initiated by pepsin, we have performed a hydrolysis experiment simulating gastric fluid for both selected L- and D-peptides. After incubation over 4 and 8 h, the sample was analysed using RP-HPLC (Supplementary Figure S8). The experiment shows that even with the increased time, the D-peptides were stable, whereas the L-peptides hydrolysis had been carried over time.

**Figure 5:**
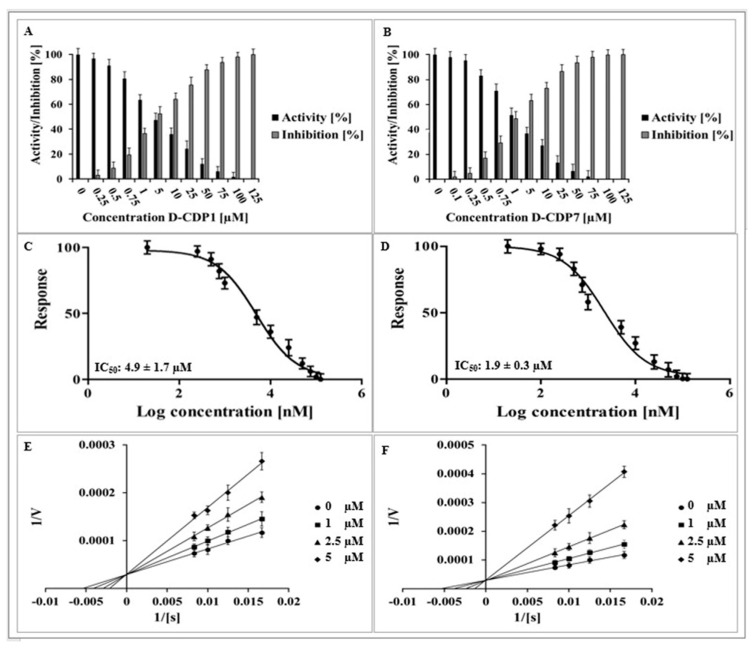
Inhibition effect, dose-response curve, and inhibition mode of D-CDP1 and D-CDP7 over SARS-CoV-2 3CL^pro^. (**A**): Normalised activity and inhibition of SARS-CoV-2 3CL^pro^ under D-CDP1 influence. (**B**): Normalised activity and inhibition of SARS-CoV-2 3CL^pro^ under D-CDP7 influence. (**C**): Dose-response curve of D-CDP1 and SARS-CoV-2 3CL^pro^. The normalised response [%] of SARS-CoV-2 3CL^pro^ is plotted against the log of the D-CDP1 concentration. (**D**): Dose-response curve of D-CDP7 and SARS-CoV-2 3CL^pro^. (**E**): Lineweaver-Burk plot for D-CDP1 inhibition of SARS-CoV-2 3CL^pro^. [S] is the substrate concentration; v is the initial reaction rate. (**F**): Lineweaver-Burk plot for D-CDP7 inhibition of SARS-CoV-2 3CL^pro^. Data shown are the mean ± SD from three independent measurements (*n* = 3).

According to the mirror symmetry, the corresponding D-enantiomer of L-CDPs should be able to bind to the L-target (3CL^pro^) with similar affinity. Regarding these facts, we have tested the selected D-peptides according to their inhibitory effect, IC_50_, inhibition mode, and K_D_ (Figure 5 and Supplementary Figure S9).

The determined IC_50_ values of D-CDP1 (4.9 ± 1.7 µM) and D-CDP7 (1.9 ± 0.3 µM) are slightly higher than that of L-peptides with an increase of two-fold for D-CDP1 (Table 3). D-CDP1 and D-CDP7 reveal the same competitive inhibition mode obtainable by the L-peptides (Figure 5E,F).

SPR experiments of the D-enantiomers with 3CL^pro^ showed no evaluable results. Therefore, the K_D_ values for the D-CDP1 and D-CDP7 interaction with 3CL^pro^ were determined using microscale thermophoresis (MST) (Supplementary Figures S9 and S10). For the sake of comparison, we also have tested L-CDP1 and L-CDP7 using the same technique. The L- and D-CDP1 and L- and D-CDP7 were labelled with CF633-NHS. The protein was titrated from 24.4 nM to 50 µM (L-CDP1, 7) and 19.5 nM to 10 µM (D-CDP1, 7). The binding of fluorescently labelled peptides to 3CL^pro^ (Supplementary Figures S9 and S10) showed the change in the thermophoretic signal, leading to K_D_ values shown in Supplementary Table S3.

A structural conversion or steric incompatibility in D-enantiomers can negatively influence the inhibition and binding behaviour of D-peptides with the target proteins [40,41] or the method of choice to determine the K_D_, which was also observed for D-CDP1 and D-CDP7. However, we have shown that both L-enantiomers (CDP1, CDP7) could be significantly modified without altering their function. The D-CDP1 interaction with 3CL^pro^ is around ten times stronger than D-CDP7; this tendency was also observed for the L-enantiomers (Table 3 and Supplementary Table S1).

### 2.6. 24 h Stability and Promiscuous Assays of L/D-CDP1 and L/D-CDP7

The term “promiscuous” inhibitors describe compounds whose inhibition mechanism involves interacting aggregates of many molecules with the target protein. Classified also as “promiscuous” are redox cycling compounds (RCCs) that generate µM concentrations of hydrogen peroxide (H_2_O_2_) in the presence of strong reducing agents, which are presented in the assay buffer in order to maintain the catalytic activity of cysteine proteases, like 3CL [42]. H_2_O_2_ generated by RCCs can indirectly inhibit the catalytic activity of proteins by oxidising accessible cysteine and or tryptophan that are present in CDP1 and CDP7 in L- and D-enantiomers.

To exclude the capacity of both D-peptides to behave as an RCC, we performed a hydrogen peroxide (H_2_O_2_) assay under the influence of TCEP. Our results demonstrated that L/D-CDP1 and L/D-CDP7 do not produce H_2_O_2_ under the influence of 1 mM TCEP and can be excluded as RCCs (Supplementary Figure S11). Furthermore, a detergent-based control was performed to exclude peptide inhibitors that possibly act as an aggregator of 3CL^pro^, and the experiment was performed by adding 0.001%, 0.01%, and 0.1% Triton X-100 to the reaction. Supposing that a molecule would exhibit significant inhibition of 3CL^pro^, which is diminished by detergent, it is almost certainly acting as an aggregation-based inhibitor, as described before [43], which was not observed for L/D-CDP1 and L/D-CDP7 (Supplementary Figure S12), discarding the potential aggregation properties of the peptides.

Many peptide-based inhibitors lose the inhibitory effect over time. The stability of the L- and D-peptides (CDP1 and CDP7) selected in this survey was tested over 24 h. The results demonstrated a constant inhibition of SARS-CoV-2 3CL^pro^ over 24 h by both peptides, demonstrating their stability over time and that they are not prone to digestion by the protease. (Supplementary Figure S13).

### 2.7. Cytotoxicity Assay of D-CDP1 and D-CDP7

**Figure 6:**
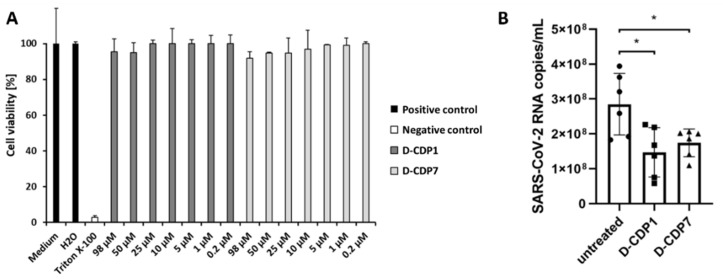
MTT assay and inhibition of SARS-CoV-2 by D-CDP1 and D-CDP7. (**A**): MTT assay of D-CDP1 and D-CDP7 on Vero CCL-81 cells. MTT assay was used to evaluate the cytotoxicity of the two D-peptides. Different concentrations of up to 98 µM were used to treat the Vero cells for 2 days. Data shown are the means ± SD from three independent measurements (*n* = 3). (**B**): Inhibition of SARS-CoV-2 by D-CDP1 and D-CDP7. Vero cells were treated with 50 µM of either D-CDP1 or D-CDP7, and after an hour of incubation, the cells were infected with SARS-CoV-2 using a multiplicity of infection (MOI) of 0.05. Untreated cells were used as control. Viral RNA in the supernatant was analysed in-house. The statistical significance of the mean values’ differences was assessed with a standard two-tailed unpaired *t*-test (demonstrated by asterisks). The graph shows individual data points with mean ± SD (*n* = 6).

A critical factor in evaluating the eligibility of potential lead peptides is cytotoxicity. The cytotoxicity of D-CDP1 and D-CDP7 conducted on the Vero cell line showed >80% viability at the Minimum Inhibitory Concentration (MIC) concerning the untreated cells (Figure 6A). The cells were treated with D-CDP1 and D-CDP7 at concentrations ranging from 0.2 to 98 µM. The results showed that both molecules have no cytotoxic effect at the calculated IC_50_ concentrations described above (Figure 6A). The low cytotoxicity of both D-peptides agrees with the results described before for the L-peptides [35].

### 2.8. Antiviral Activity of D-CDP1 and D-CDP7

To further substantiate the enzyme inhibition results, we evaluated the ability of these peptides to inhibit SARS-CoV-2. To test whether the two SARS-CoV-2 3CL^pro^ inhibitors, D-CDP1 and D-CDP7, could inhibit SARS-CoV-2 replication in cell culture, an African green monkey cell line that supports productive SARS-CoV-2 replication was used. These Vero cells were pre-treated with the two inhibitors for 1 h; subsequently, the cells were infected at a MOI of 0.05. Viral replication was analysed by determining the SARS-CoV-2 RNA within the cell culture supernatant 2 dpi (days post-infection). Pre-treatment at a non-toxic concentration (50 µM) resulted in a significant decrease in viral RNA after 2 dpi (Figure 6B), suggesting both SARS-CoV-2 3CL^pro^ inhibitors, D-CDP1 and D-CDP7, contribute to impaired SARS-CoV-2 replication through their anti-3CL^pro^ activity.

These experiments showed that D-peptides could impair SARS-CoV-2 replication, most likely by explicitly targeting the viral protease 3CL^pro^. While a twofold reduction in viral RNA, as seen after treatment with D-CDP1, may not seem much regarding the high replication rate, it clearly shows that our compounds exert antiviral activity within SARS-CoV-2-infected cells. These results provide the first proof that the peptides target the inhibition of the SARS-CoV-2 replication. Whether this effect is due to the anti-3CL^pro^ activity and whether the inhibitory effect observed can be optimised by additional modifications is subject to further investigation.

Future work should aim to reveal the mechanism behind the action of both D-peptides and the optimisation of the amino acid composition.

### 2.9. Structural Interfaces of the Predicted 3CL^pro^/L-CDP1 Complex

The 3D model of the L-CDP1 and L-CDP7 peptides was generated using I-TASSER server [44], which corroborates the CD spectroscopy results. Based on the mode of inhibition studies, protein-peptide (L-CDP1 and L-CDP7) docking was performed applying to the HADDOCK web server program. The atomic coordinates of SARS-CoV-2 3CL^pro^ (PDB entry: 6M2Q) and the atomic coordinates of the peptides were submitted to the platform. The selected 3CL^pro^/peptide complex was subject to 100 ns (3 replicas) of molecular dynamics simulation (MD) embedded in an octahedral TIP3P water box. The three replicas were simulated using the same starting atom position with different random initial velocities. As individual chains form the two active sites of the 3CL^pro^ homodimer, MD simulations were carried out with monomeric 3CL^pro^ in complex with both peptides, separately. The flexibility of the protease/peptides structure of the MD simulation system was monitored by calculating the RMSD, RMSF, RoG, and the surface area (Supplementary Figure S14). Three replicates (100 ns) of MD simulation were conducted with 3CL^pro^/L-CDP1 and L-CDP7 to assess the time stability of the binding mode previously proposed. The trajectory was evaluated by observing the deviation values. A significant deviation was observed for the backbone atoms of the 3CL^pro^ plus L-CDP1 and 3CL^pro^ plus L-CDP7 along the 100 ns of MD (Supplementary Figure S15). Large motions of the single promoter of the 3CL^pro^ cause such deviations. On the other hand, RMSF shows a relatively low fluctuation and H-bonds profiles (Supplementary Figure S15), indicating that the L-CDP1 keeps forming favourable interactions with 3CL^pro^ residues during simulation time.

**Figure 7:**
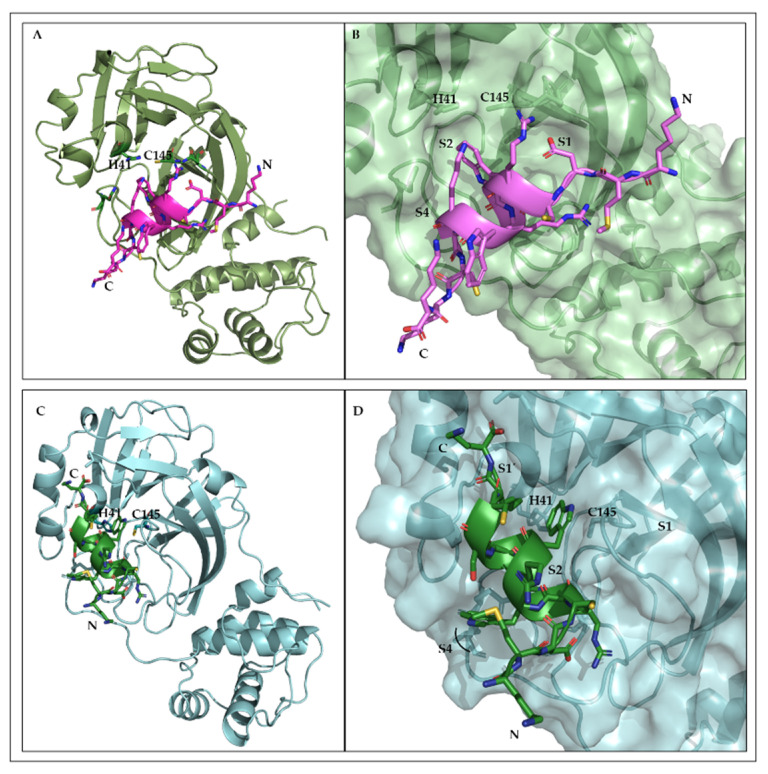
SARS-CoV-2 3CL^pro^ binding site. (**A**): Cartoon representation of the protease in complex with the L-CDP1 peptide (cartoon/sticks); the side chain of the catalytic residues His41 and Cys145 are shown in sticks representation. (**B**): Surface representation of the 3CL^pro^ complex with the L-CDP1 peptide (cartoon/sticks). The interaction interface of the peptide with the substrate-binding sites, which is blocked by the peptide, is shown according to S1, S2, and S4. (**C**): Cartoon representation of the protease in complex with the L-CDP7 peptide (cartoon/sticks); the side chain of the catalytic residues His41 and Cys145 are shown in sticks representation. (**D**): Surface representation of the 3CL^pro^ complex with the L-CDP7 peptide (cartoon/sticks). The interaction interface of the peptide with the substrate-binding sites that are blocked by the peptide is shown according to S1’, S2, and S4.

The analysis of the 3CL^pro^/L-CDP1 and 3CL^pro^/L-CDP7 complexes provide valuable information on the interaction interfaces. Structural representation (Figure 7) shows that L-CDP1 extends along with the S1, S2, and S4 substrate binding sites. On the other hand, L-CDP7 interacts with the substrate-binding sites S1’, S2, and S4. The substitution of the amino acid residue Cys10 with Ser residue shows an evident change in how the peptide interacts with the protein. Structural analysis of L-CDP1 shows that Cys10 forms a hydrogen bond with Trp6, essential for maintaining the peptide structure. In L-CDP1, the Trp6 at P2 and Cys10 at P4 insert into well-defined pockets (Figure 7B). Our previous work (Hernández et al., 2022) demonstrates the preference for aromatic residue in the S2 pocket of 3CL^pro^ [45]. Even though Trp6 in the L-CDP-7 peptide does not show any interaction with amino acids at the S4 site, the new coordinate in the interaction of the peptide with the protein allows Trp8 to establish contact with amino acids at the P2 site, thus confirming the preference of the S2 site for aromatic residue. The S1 pocket accommodates the Asp3 residue in L-CDP1; however, L-CDP7 does not show any interaction with the S1 site. Asn142, which is demonstrated to be a point of conformational plasticity, interacts through an H-bond with the Arg5 residue of the peptide L-CDP1 and by H-bonds with amino acids residues of the peptide L-CDP7 (Cys4 and Arg5), validating that the side-chain of Asn142 can assume conformations in the solution that favour interaction to the ligands. The structural analyses demonstrate that L-CDP1 and L-CDP7 displayed good complementarity with the 3CL^pro^ binding site (Figure 7) across the access of the substrate, which was also shown by the in vitro test.

MD simulation revealed a possible mode of interaction of the L-CDP1 peptide with SARS-CoV-2 3CL^pro^. Therefore, the amino acid residue of the active site does not interact directly with the peptide; however, the L-CDP1 is accommodated in the substrate-binding regions (Figure 7B), confirming the competitive mode of interaction already described by experimental results (Figure 3A).

## 3. Materials and Methods

### 3.1. Crotamine Purification

The purification of crotamine has been described previously [46]. Briefly, crotamine from crude *Crotalus durissus terrificus* venom obtained from CEVAP (Center for the Study of Venoms and Venomous Animals), Botucatu, Brazil, was isolated by a single cation-exchange chromatography step by using a MonoS HR 10/10 column (Amersham Biosciences, Amersham, United Kingdom).

### 3.2. Crotamine Derivative Peptides (CDP) Synthesis

Synthetic crotamine derivative peptides (CDP) in the L- and D-enantiomeric conformations were synthesised by Genscript (Leiden, NL, USA), with a purity of >95% (Supplementary Figure S16). The peptides were acetylated at the N-terminus and methylated at the C-terminus. Essential information about the CDPs used in this study is summarised in Supplementary Figures S2 and S3 and Table S2.

### 3.3. Cloning, Expression and Purification of SARS-CoV-2 3CL^pro^

SARS-CoV-2 3CL^pro^ (Uniprot entry: P0DTD1, virus strain: hCoV-19/Wuhan/WIV04/2019) was cloned, expressed, and purified, as described previously [31].

### 3.4. Activity Assay of SARS-CoV-2 3CL^pro^

SARS-CoV-2 3CL^pro^ activity assay was performed using a fluorogenic substrate DABCYL-KTSAVLQ↓SGFRKME-EDANS (Bachem, Bubendorf, Switzerland) in a buffer containing 20 mM Tris pH 7.2, 200 mM NaCl, 1 mM EDTA, and 1 mM TCEP [31,32,33,34]. The reaction mixture was pipetted in a Corning 96-well plate (Merck, Darmstadt, Germany) containing 0.5 µM protein. The assay was initiated with the addition of the substrate at a final concentration of 50 µM. The fluorescence intensities were measured at 60 s intervals over 30 min using an Infinite 200 PRO plate reader (Tecan, Männedorf, Switzerland), and the temperature was set to 37 °C. The excitation and emission wavelengths were 360 and 460 nm, respectively.

### 3.5. Inhibition Assay of SARS-CoV-2 3CL^pro^

The activity assay described above investigated the inhibition of SARS-CoV-2 3CL^pro^ activity by Cro, L- and D-CDPs. 30 µM of the peptides was used for a preliminary screening test.

For the final inhibition assays, 0.5 µM of the protein was mixed with 0–300 µM Cro, 0–150 µM L-CDPs, and 0–125 µM D-CDPs and subsequently incubated for 30 min at RT. An additional experiment was conducted where the substrate and the inhibitor were added simultaneously (without incubation). When the substrate with a final concentration of 50 µM was added to the mixture, the fluorescence intensities were measured at 60 s intervals over 30 min using an Infinite 200 PRO plate reader (Tecan, Männedorf, Switzerland). The temperature was set to 37 °C, and the excitation and emission wavelengths were 360 and 460 nm, respectively. Inhibition assays were performed in triplicate.

The IC_50_ value was calculated by plotting the initial velocity against various concentrations of the combined molecules using a dose-response curve in GraphPad Prism5 software (Tecan, Männedorf, Switzerland). Data are presented as mean ± SD.

### 3.6. Determination of Inhibition Mode

The inhibition mode was determined using different final concentrations of the inhibitors and substrate. The L-peptides with the most potent inhibition effect were chosen for this analysis, as were the selected D-peptides: L/D-CDP1, L/D-CDP7, and L-CDP8. The remaining L-peptides were not tested.

Briefly, 0.5 µM SARS-CoV-2 3CL^pro^ was incubated with the inhibitor, in various concentrations, for 30 min at RT. Subsequently, the reaction was initiated by adding the corresponding concentration series of the substrate. The data were analysed using a Lineweaver-Burk plot; therefore, the reciprocal of velocity (1/V) vs the reciprocal of the substrate concentration (1/[S]) was compared [47,48]. All measurements were performed in triplicate, and data are presented as mean ± SD.

### 3.7. Inhibitor Stability over 24 h

Stable inhibition of SARS-CoV-2 3CL^pro^ by L-/D-CDP1 and L-/D-CDP7 was monitored via a 24 h inhibition experiment. Briefly, 0.5 µM SARS-CoV-2 3CL^pro^ was incubated with 5 µM of the peptide and incubated for 1/2 h, 1 h, 2 h, 3 h, 4 h, 5 h, and 24 h at RT. The control was performed with the 3CL^pro^ in the absence of the peptide and measured together after each time point. Subsequently, the reaction was initiated by the addition of the substrate. All measurements were performed in triplicate, and data are presented as mean ± SD.

### 3.8. Assays to Exclude L-CDPs and D-CDPs as Promiscuous Inhibitors

A detergent-based control assay was performed to exclude inhibitors that possibly act as aggregators of the 3CL^pro^ by adding 0.001%, 0.01%, and 0.1% of Triton X-100 to the reaction [43]. Four concentrations of L-CDP1 and D-CDP1 (0.5 µM, 1 µM, 5 µM, and 10 µM) and L-CDP7 and D-CDP7 (0.25 µM, 0.5 µM, 1 µM, and 5 µM), were tested. All measurements were performed in triplicate, and data are presented as mean ± SD.

Redox cycling compounds (RCCs) generate H_2_O_2_ in the presence of potent reducing agents (e.g., DTT or TCEP). We performed a colourimetric assay to exclude L-CDP1, D-CDP1 and L-CDP7, D-CDP7 as a compound that induces redox cycling in reducing environments.

The assay was performed in Nunc 96-well plates with a flat bottom (Thermofisher Scientific, Waltham, MA, USA), and the final volume was 60 µL. In summary, 0–60 µM L-CDP1, D-CDP1 and L-CDP7, D-CDP7 were tested in the same activity assay buffer (as described above), and 1 mM TCEP was added separately. HRP-PR and 100 µM H_2_O_2_ were used as control. The HRP-PR detection reagent (100 µg/mL phenol red and 60 µg/mL HRP, final) was prepared in Hank’s balanced salt solution (HBSS). HPR-PR and PR without the addition of H_2_O_2_ were used as negative controls. The reducing agents could mediate the oxidation of phenol red (PR) (Merck, Darmstadt, Germany) based on the H_2_O_2_-dependent horseradish peroxidase (HRP (Merck, Darmstadt, Germany)) mediated oxidation, which produces a change in its absorbance at 610 nm in alkaline pH [42]. Next, L-CDP1, D-CDP1 and L-CDP7, D-CDP7 were incubated with TCEP at RT for 30 min before adding the HRP-PR detection reagent. After an additional incubation period at RT (10 min), the assay was terminated by adding 10 µL of 1N NaOH to all wells. The absorbance of the phenol red was measured at 610 nm using an Infinite 200 PRO plate reader (Tecan, Männedorf, Switzerland). All measurements were performed in triplicate, and data are presented as mean ± SD.

### 3.9. L- and D-Peptide Stability against Pepsin Hydrolysis

The preparation of the pepsin solution was performed as described before [49,50]. The experiment solution contains 2 mg/mL NaCl, 3.2 mg/mL pepsin, and 80 mM HCL at a pH 1.0. This solution simulated gastric fluid. For the stability tests, 150 µM RD2 [50] has been used as a control, and L/D-CDP1 and L/D-CDP7 were incubated with the pepsin solution at 37 °C and 500 rpm for 8 h. Samples were taken after 0 h, 4 h, and 8 h. The samples were analysed by reversed-phase high-performance liquid chromatography (RP-HPLC). The RP-HPLC system (Agilent Technologies, Santa Clara, CA, USA; 1200 series) consisted of a manual injector, quaternary pump, a thermostatted column compartment, and a variable wavelength detector. Chromatography was performed with a C18 column (Agilent Technologies, Santa Clara, CA, USA; ZORBAX 300SB-C18 5 µm, 4.6 × 250 mm) at 25 °C and 214 nm with a flow rate of 1 mL/min. The sample injection volume was 20 µL. Chromatograms were recorded and analysed with the Agilent software ChemStation (G2175BA; B03.01). Mobile phases were acetonitrile (A) and water (B), each supplemented with 0.15% trifluoroacetic acid (TFA) (*v*/*v*). The samples were measured isocratically at 10% solvent A for 30 min.

### 3.10. Determination of Dissociation Constant Using Surface Plasmon Resonance

The dissociation constant (K_D_) of L-enantiomeric peptides CDP1 and CDP7 binding to SARS-CoV-2 3CL^pro^ was determined by Surface Plasmon Resonance Spectroscopy (SPR) using a Biacore 8K instrument (GE Healthcare, Uppsala, Sweden). The peptides were immobilised on two separate channels on a series S CM-5 sensor chip (Cytiva, Uppsala, Sweden) by amine coupling. Both flow cells on each channel were activated by a mixture of 50 mM N-Hydroxysuccinimide (NHS) and 16.1 mM N-ethyl-N’-(dimethylaminopropyl) carbodiimide (EDC) (XanTec, Düsseldorf, Germany) for 7 min. The peptides were diluted to 50 µg/mL in 10 mM sodium acetate pH 5 (Merck, Darmstadt, Germany) and injected in overflow cell two of each channel to a final signal of 3400 RU for L-CDP1 and 1300 RU for L-CDP7. After the peptides were immobilised, each channel’s ligand and reference flow cells were quenched by a 7-min injection of 1 M ethanolamine pH 8.5 (XanTec, Düsseldorf, Germany).

The K_D_ multicycle experiments were determined with PBS containing 0.05% Tween 20 (AppliChem, Darmstadt, Germany) pH 7.4 as the running buffer. The temperature was set at 25 °C with a flow rate of 30 µL/min. Diluted 3CL^pro^ in the running buffer to the desired concentrations range between 2000 nM–0.9 nM using 1:2 dilution steps. All samples were injected over the flow cells for 180 s, followed by a dissociation phase of 900 s with a running buffer. A regeneration step was performed to ensure complete dissociation of the protease from the peptides, with a 45 s injection of a mixture containing 50 mM oxalic acid, 50 mM phosphoric acid, 50 mM formic acid, and 50 mM malonic acid at pH 5. The reference flow cell and buffer injections (c = 0 nM) were used to double reference the sensorgrams. The sensorgrams were fitted by the Steady-State Affinity model implemented in the Biacore Insight Evaluation Software for data evaluation. The experiments were performed in duplicate, and the results are shown as the mean ± STD.

### 3.11. Determination of Dissociation Constant Using Microscale Thermophoresis (MST)

The binding affinity of the L- and D-enantiomeric peptides CDP1 and CDP7 towards SARS-CoV-2 3CL^pro^ were determined using Microscale thermophoresis (MST). Following the manufacturers’ instructions, peptides were first fluorescently labelled with CF633-NHS (Sigma, Darmstadt, Germany). In short, a 100 µmol/L peptide solution was incubated with 500 µmol/L CF633-NHS solutions in 100 mmol/L NaHCO_3_ pH 8.3 at 25 °C for 1 h with slight agitation. After the reaction was completed, the samples were purified using reversed-phase liquid chromatography on an Agilent 1200 system (Agilent Technologies, Santa Clara, CA, USA). The samples were loaded onto a Zorbax SB-300 C-18 column (4.6 ∗ 250 mm, 5 µm) (Agilent Technologies, Santa Clara, CA, USA). Mobile phases consisted of A: H_2_O + 0.1% Trifluoroacetic Acid (TFA) (Sigma, Darmstadt, Germany) and B: Acetonitrile (Roth, Karlsruhe, Germany) + 0.1% TFA. A gradient from 15% B to 45% B in 20 min was applied at a flow rate of 1 mL/min, and CF633 labelled peptides were eluted after 9.2 min, collected, and lyophilised (Christ, Alpha 2–4 LSCbasic, Christ, Osterode am Harz, Germany) (Supplementary Figure S17).

Lyophilised CF-633 labelled peptides were dissolved in 100 µL H_2_O. The absorbance of the sample was measured at 280 and 633 nm using a Shimadzu UV 1800 instrument (Shimadzu, Duisburg, Germany) to calculate the concentration of the peptide.

The MST measurement was performed using a serial dilution of the protease (3CL) (10 µmol/L to 19.53 nmol/L) with a 1:1 dilution step in PBS + 0.05% Tween 20. To each sample, CF633-peptide was added to a final concentration of 100 nmol/L. The measurements were performed on a Nanotemper Monolith NT.115 instrument (NanoTemper Technologies, Munich, Germany). For CF-633 as the fluorescent dye, a red LED was used at 20% power. The MST power was set to 40%, and the temperature was kept constant at 25 °C. Data were evaluated with thermophoresis with T-Jump evaluation, with the cold region from −1 s to 0 s and the hot region from 20.67 s to 21.67 s. Data were fitted to the K_D_ Model integrated into the analysis software. The experiments were performed in triplicate, and the results are shown as the mean ± STD.

### 3.12. Circular Dichroism (CD) Spectroscopy

CD measurements were carried out with a Jasco J-1100 Spectropolarimeter (Jasco, Germany). Far-UV spectra were measured at 190 to 260 nm using a peptide concentration of 30 µM in H_2_O. The secondary structure of L-CDP1, L-CDP2, L-CDP7, and L-CDP8 and D-CDP1 and D-CDP7 was checked. A 1 mm path length cell was used for the measurements; 15 repeat scans were obtained for each sample, and five scans were conducted to establish the respective baselines. The averaged baseline spectrum was subtracted from the averaged sample spectrum. The results are presented as molar ellipticity [θ], according to Equation (1):

[θ]λ = θ/(c × 0.001 × l × n)(1)

where θ is the ellipticity measured at wavelength λ (deg), c is the peptide concentration (mol/L), 0.001 is the cell path length (cm), and n is the number of amino acids. Secondary structure prediction based on the CD data was performed with the BeStSeL online tool [51].

### 3.13. Cell Viability Assay

The cell viability assay was performed using the reduction of [3-(4,5-dimethylthiazol-2-yl)-2,5-diphenyl tetrazolium bromide—MTT] to investigate the cytotoxicity of D-CDP1 and D-CDP7. Therefore, Vero cells were cultivated in DMEM medium supplemented with 2% fetal calf serum (FCS) and 1% penicillin/streptomycin. A total of 2000 cells per well in a volume of 100 µL were seeded on flat-bottomed 96-well plates (VWR, Radnor, USA) and incubated in a 95% humidified atmosphere with 5% CO_2_ at 37 °C for 24 h. Subsequently, the cells were treated with 0.2, 1, 5, 10, 25, 50, and 98 µM of each molecule at 37 °C with 5% CO_2_ for 24 h.

According to the manufacturer’s instructions, cell viability was measured using the Cell Proliferation Kit I (Roche, Basel, Switzerland) in a 3-fold determination. The absorbance of the formazan product was determined by measuring the absorption at 570 nm and subtracted by the absorbance at 690 nm in a microplate reader (BMG Labtech, Ortenberg, Germany). The results were normalised to the mean value of cells treated with medium only. As a negative control for cytotoxicity, 0.1% Triton X-100 diluted in the medium was used. Cell viability was calculated according to Equation (2):

(T/C) × 100%(2)

where T and C represented the optical density of the treated well and control groups, respectively. The MTT assays for D-CDP1 and D-CDP-7 were performed in triplicate, and the results are shown as mean ± SD.

### 3.14. SARS-CoV-2 Infection Assay

Experiments with SARS-CoV-2 were performed under biosafety level 3 (BSL-3) conditions at the University Hospital Düsseldorf. Vero cells (ATCC-CCL-81, obtained from LGC Standards) were seeded into the wells of a 96-well plate in Dulbecco’s Modified Eagle Medium—DMEM (Gibco, Carlsbad, CA, USA) with 2% fetal calf serum (PAN Biotech, Germany) and 1% penicillin/streptomycin (Gibco, Carlsbad, CA, USA) at a density of 5 × 103 cells well-1. Following overnight incubation, the medium (100 µL) was replaced with a 100 µL new medium containing the respective compounds D-CDP1 or D-CDP7 at a final concentration of 50 µM, and the cells were incubated for one hour at 37 °C in a humidified cell culture incubator. Cells were infected with a SARS-CoV-2 B1.1 isolate (SARS-CoV-2 NRW-42 isolate; GISAID accession number: EPI_ISL_425126) (PMID: 32876341 [52]) at a multiplicity of infection (MOI) of 0.05. At two days post-infection (dpi), quantitative RT-PCR was performed. Therefore, 50 µL cell culture supernatant was mixed with 200 µL AVL buffer (Qiagen, Germany) following 10 min of incubation at RT. Subsequently, 200 µL of 100% ethanol was added to the mixture. Following the manufacturer’s instructions, RNA was extracted from 200 µL of the inactivated supernatant using the EZ1 Virus Mini Kit (Qiagen, Germany). As described previously, the 60 µL eluate was analysed via in-house qRT-PCR [52]. In short, a 113 bp sequence of the SARS-CoV-2 Envelope open reading frame was amplified using the real-time TaqMan technique.

### 3.15. Statistical Analysis

All data are expressed as the mean ± the standard deviations (SDs). The statistical significance of the mean values’ differences was assessed with one-way analyses of variance (ANOVA), followed by Tukeys’ multiple comparison test. Significant differences were considered at *p* < 0.05 (*), *p* < 0.01 (**), and *p* < 0.001 (***). A standard two-tailed unpaired *t*-test was used to analyse the inhibitory effect of D-CDP1 and D-CDP7 on SARS-CoV-2 replication. All statistical analyses were performed with GraphPad Prism software version 8 (San Diego, CA, USA).

### 3.16. System Preparation

The 3D structure of the SARS-CoV-2 3CL protease was retrieved from the PDB databank (PDB code: 6M2Q), and the L-CDP1 3D model was generated using I-TASSER server [44]. Docking was performed using the HADDOCK web server [53]. The top four structures were downloaded and viewed by PyMOL. The H++ web server [54] was used to assign the correct lateral chain protonation of the amino acids to pH 7.4 for the simulated systems. After that, the complex (3CL^pro^/L-CDP1) was placed in an octahedral TIP3P water box with water extended 10 Å away from any solute atom and Cl− counter-ion was used to neutralise the system.

### 3.17. Simulation Setup

MD simulations were performed using Amber18 [55]. Atomic interactions were represented by the FF19SB [56] force field. Starting structures were submitted in a two-step minimisation process to remove bad contacts. In the first stage, the complex was restricted and minimised during the 5000 steepest descending steps, followed by 5000 conjugated gradient steps and carried out in harmonic restraints (k = 10 kcal∙mol^−1^ Å^2^). The second round of unconstrained energy minimisation was performed during 10,000 steps. The system was slowly heated using a linear temperature gradient from 0 to 298 K for 500 ps under a constant atom number, volume, and temperature (NVT) ensemble. The complex was constrained with a constant force of 10 kcal/mol Å^2^. Subsequently, the equilibration step was performed using an isothermal−isobaric (NPT) ensemble for 5 ns, with decreasing restrained force constant from 10 to 0 kcal/mol Å^2^. Lastly, the production run was performed for 100 ns in the NVT ensemble without any restriction. The temperature (298 K) and pressure (1 bar) were controlled by Langevin coupling. Three replicas were simulated using the same starting atom position with different random initial velocities, according to the Maxwell-Boltzmann distribution. The SHAKE constraints were applied to all atoms involving H-bonds to allow a 2-fs dynamic time step. Long-range electrostatic interactions were calculated by the particle-mesh Ewald method (PME) using the 8-Å cutoff [57].

### 3.18. Molecular Dynamics Analysis and Interaction Energy Calculation

MD results were analysed using CPPTRAJ [58] tools for the AmberTools19 package [59]. Root-Mean-Square Deviation (RMSD) was used to investigate the equilibration and convergence of the simulations. The Root-Mean-Square Fluctuation (RMSF) of the Cα atoms accessed protein and peptide flexibility. Structural changes in the protein were evaluated by the radius of gyration (RoG) and solvent accessible surface area.

The molecular mechanics/generalised Born surface area (MM/GBSA) was calculated between the protein–peptide complex using the generalised Born (GB)-Neck2 [60] implicit solvent model (igb = 8) in the steady-state regime of the entire simulation time, stripping the solvent and ions. Root mean square fluctuations (RMSFs) were calculated using the rmsf command of cpptraj [61]. Finally, hydrogen bonds (H-bonds) formed at the complex interfaces during the MD simulations were determined with hbond command of cpptraj with default geometric parameters [61].

## 4. Conclusions

Snake venom components have shown great potential for developing lead compounds for new drugs and can be considered mini-drug libraries. For instance, Captopril (Enalapril), Integrilin (Eptifibatide), and Aggrastat (Tirofiban) are FDA-approved drugs based on snake venoms. Defibrase/Reptilase (Batroxobin), Hemocoagulase, and Exanta (Ximelagatran) are not clinically approved in the US but have been approved for use in other countries. Several venom-derived drugs are involved in preclinical or clinical trials for a variety of therapeutic applications. Our results demonstrated the promising uses of peptide inhibitors (D-CDP1 and D-CDP7) designed from a polypeptide of snake venom. The advantage of the selected wild types was because of their cell penetration properties, even in D-enantiomer form, high stability and specificity, and selectivity against the target 3CL protease. The analysis of the interactions occurring at the 3CL^pro^/L-CDP1 complex provides valuable information to study the 3CL^pro^/D-CDP peptides interface. However, in particular, more investigation into the unquestionable, untapped therapeutic potential of the selected D-peptide is still required and future optimisation.

## Figures and Tables

**Table 1:** Summary of the SARS-CoV-2 3CL^pro^ inhibition experiments by crotamine and CDPs.

Molecule	Sequence	IC50 [µM] ± STD	Inhibition Type ^1^
Crotamine		40 ± 3.1	−
L-CDP1	KMDCRWRWKCCKK	1.9 ± 0.3	Competitive
L-CDP2	KMDSRWRWKSSKK	5.0 ± 0.8	−
L-CDP3	KMDCRWRWKSSKK	8.5 ± 1.0	−
L-CDP4	KMDSRWRWKCCKK	5.1 ± 1.1	−
L-CDP5	KMDSRWRWKSCKK	6.2 ± 0.5	−
L-CDP6	KMDSRWRWKCSKK	7.5 ± 0.7	−
L-CDP7	KMDCRWRWKSCKK	1.5 ± 0.4	Competitive
L-CDP8	KMDCRWRWKCSKK	2.1 ± 0.4	Competitive
L-CDP9	RWRWKCCKK	4.9 ± 0.6	−

^1^ Inhibition mode was determined for L-CDP1, L-CDP7, and L-CDP8 due to their most substantial inhibition against 3CL^pro^. The inhibition mode of the other L-Peptides was not determined (−).

**Table 2:** SARS-CoV-2 3CL^pro^ SPR experiments by L-CDP1 and L-CDP7.

Molecule	Sequence	K_D_ [nM] ± STD
L-CDP1	KMDCRWRWKCCKK	65 ± 20.1
L-CDP7	KMDCRWRWKSCKK	304 ± 70.3

The experiments were performed in duplicate, and the results are shown as the mean ± STD. The K_D_ values of the single experiments are shown in Supplementary Table S2.

**Table 3:** Affinity and Inhibitory Activity of the D-CDP1 and D-CDP-7 Peptides.

Molecule	IC_50_ [µM] ± STD	Inhibition Type	K_D_ [nM] ± STD
D-CDP1	4.9 ± 1.7	Competitive	185.7 ± 17.8
D-CDP7	1.9 ± 0.3	Competitive	1951.3 ± 87.5

The MST experiments were performed in triplicate, and the results are shown as the mean ± STD. The K_D_ values of the single experiments are shown in Supplementary Table S3.

## Data Availability

Data is contained within the article and supplementary material.

## Supplementary Materials

The following supporting information can be downloaded at https://www.mdpi.com/article/10.3390/ph15050540/s1, Figure S1: Purification of SARS-CoV-2 3CL^pro^; Figure S2: Primary structure of L-CDP1 to L-CDP5; Figure S3: Primary structure of L-CDP6 to L-CDP9; Figure S4: Inhibition effect of Crotamine and L-CDPs over SARS-CoV-2 3CL^pro^; Figure S5: Crotamine and L-CDPs with inhibitory activity against SARS-CoV-2 3CL^pro^; Figure S6: Circular dichroism (CD) spectroscopy of L-CDP1, L-CDP2, L-CDP7, and CDP8; Figure S7: Dissociation constant (K_D_) determination of L-CDP1 and L-CDP7 binding to SARS-CoV-2 3CL^pro^ using surface plasmon resonance (SPR); Figure S8: Stability of L- and D-CDPs against Pepsin hydrolysis; Figure S9: K_D_ determination of D-CDP1 and D-CDP7 binding to SARS-CoV-2 3CL^pro^ using MST; Figure S10: K_D_ determination of L-CDP1 and L-CDP7 binding to SARS-CoV-2 3CL^pro^ using MST; Figure S11: H_2_O_2_ generating capacity of L- and D-CDP1, CDP7 under the influence of 1 mM TCEP; Figure S12: Effect of Triton X-100 on the L- and D-CDP1, CDP7 inhibition against SARS-CoV-2 3CL^pro^; Figure S13: 24 h inhibition experiment of L- and D-CDP1, CDP7 against SARS-CoV-2 3CL^pro^. Figure S14: Time-dependent modifications of the 3CL^pro^/L-CDP1 and L-CDP-7 complexes; Figure S15: Intermolecular H-bonds of the replicates MD simulations; Figure S16: HPLC chromatogram of the L-CDP and D-CDP peptides; Figure S17: HPLC chromatogram of the L/D-CDP1 and L/D-CDP7 peptides after the labeling process with CF633-NHS; Table S1: K_D_ determination of L-CDP1 and L-CDP7 using SPR; Table S2: Basic information about tested L- and D-CDPs; Table S3: K_D_ determination of L/D-CDP1 and L/D-CDP7 using MST.
