# Supplementary material for: Design of D-Amino Acids SARS-CoV-2 Main Protease Inhibitors Using the Cationic Peptide from Rattlesnake Venom as a Scaffold
Source: Pharmaceuticals (Basel). 2022 Apr 27;15(5):540. doi: 10.3390/ph15050540 (PMC9146215; doi:10.3390/ph15050540)
Supplement: Supplementary file 1 [file pharmaceuticals-15-00540-s001.zip › pharmaceuticals-1638194-supplementary.pdf]

Supplementary Information

Design of L and D-amino acids SARS-CoV-2 3CL protease inhibitors using the cationic peptide from rattlesnake venom as a scaffold

Raphael J. Eberle[1,2], Ian Gering[1], Markus Tusche[1], Philipp N. Ostermann[3], Lisa Müller[3], Ortwin Adams[3], Heiner Schaal[3], Danilo S. Olivier[4], Marcos S. Amaral[5], Raghuvir K. Arni[6], Dieter Willbold[1,2,7] Mônika A. Coronado[1,*]

[1]Institute of Biological Information Processing (IBI-7: Structural Biochemistry), Forschungszentrum Jülich, Jülich, Germany.

[2]Institut für Physikalische Biologie, Heinrich-Heine-Universität Düsseldorf, Universitätsstraße, Düsseldorf, Germany.

[3]Institute of Virology, Medical Faculty, University Hospital Düsseldorf, Heinrich-Heine-Universität, Düsseldorf, Germany.

[4]Campus Cimba, Federal University of Tocantins, Araguaína, TO 77824-838, Brazil.

[5]Institute of Physics, Federal University of Mato Grosso do Sul, Campo Grande, MS 79070-900, Brazil

[6]Multiuser Center for Biomolecular Innovation, IBILCE, Universidade Estadual Paulista (UNESP), São José do Rio Preto-SP, Brazil.

[7]JuStruct: Jülich Centre for Structural Biology, Forschungszentrum Jülich, Jülich, Germany.

* Correspondence to m.coronado@fz-juelich.de

Table of contents

Figure S1. Purification of SARS-Cov-2 3CL^{pro}.
Figure S2. Primary structure of L-CDP1 to L-CDP5.
Figure S3. Primary structure of L-CDP6 to L-CDP9.
Figure S4. Inhibition effect of Crotamine and L-CDPs over SARS-CoV-2 3CL^{pro}.
Figure S5. Crotamine and L-CDPs with inhibitory activity against SARS-CoV-2 3CL^{pro}.
Figure S6. Circular dichroism (CD) spectroscopy of L-CDP1, L-CDP2, L-CDP7 and CDP8.
Figure S7. K_D determination of L-CDP1 and L-CDP7 binding to SARS-CoV-2 3CL^{pro} using SPR.
Figure S8. Stability of L- and D-CDPs against Pepsin hydrolysis.
Figure S9. K_D determination of D-CDP1 and D-CDP7 binding to SARS-CoV-2 3CL^{pro} using MST.
Figure S10. K_D determination of L-CDP1 and L-CDP7 binding to SARS-CoV-2 3CL^{pro} using MST.
Figure S11. H_2O_2 generating capacity of L- and D-CDP1, CDP7 under the influence of 1 mM TCEP.
Figure S12. Effect of Triton X-100 on the L- and D-CDP1, CDP7 inhibition against SARS-CoV-2 3CL^{pro}.
Figure S13. 24 h inhibition experiment of L- and D-CDP1, CDP7 against SARS-CoV-2 3CL^{pro}.
Figure S14. Time-dependent modifications of the 3CL^{pro}/L- D-CDP1 complex.
Figure S15. Intermolecular H-bonds of the replicates MD simulations.
Figure S16. HPLC chromatogram of the L-CDP and D-CDP peptides.
Figure S17. HPLC chromatogram of the L/D-CDP1 and L/D-CDP7 peptides after the labeling process with CF633-NHS.

Table S1. K_D determination of L-CDP1 and L-CDP7 using SPR.
Table S2. Basic information about tested L- and D-CDPs.
Table S3. K_D determination of L/D-CDP1 and L/D-CDP7 using MST.

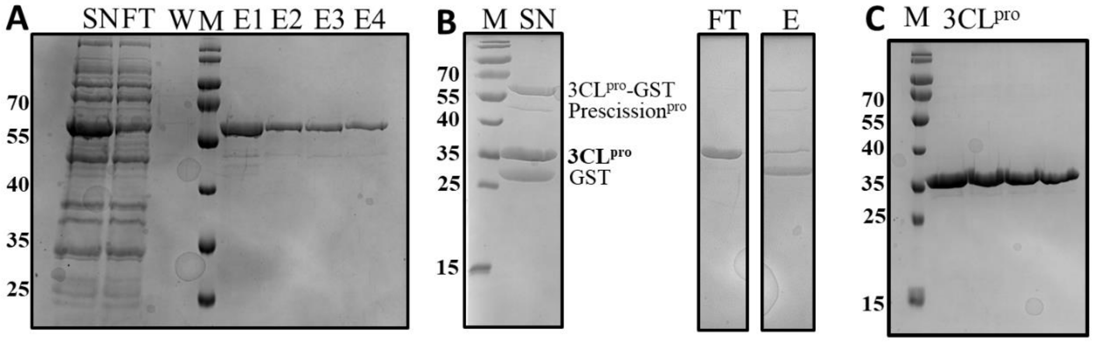

Figure S1. Purification of SARS-Cov-2 3CL^{pro}. **A:** SDS 15% Gel of 3CL^{pro}_GST purification with GSH sepharose. SN: supernatant, FT: flow-through, W: washing step, M: protein marker, E1-E4: Elution. **B:** SDS 15% Gel of the 3CL^{pro}_GST fusion protein cleavage by PreScission^{pro}. M: protein marker. SN: cleaving approach, containing 3CL^{pro}_GST, PreScission^{pro}, 3CL^{pro} and GST. FT: contain 3CL^{pro}, E: elution step including uncleaved 3CL^{pro}_GST, PreScission^{pro} and GST tag. **C:** SDS 15% Gel of pure 3CL^{pro}.

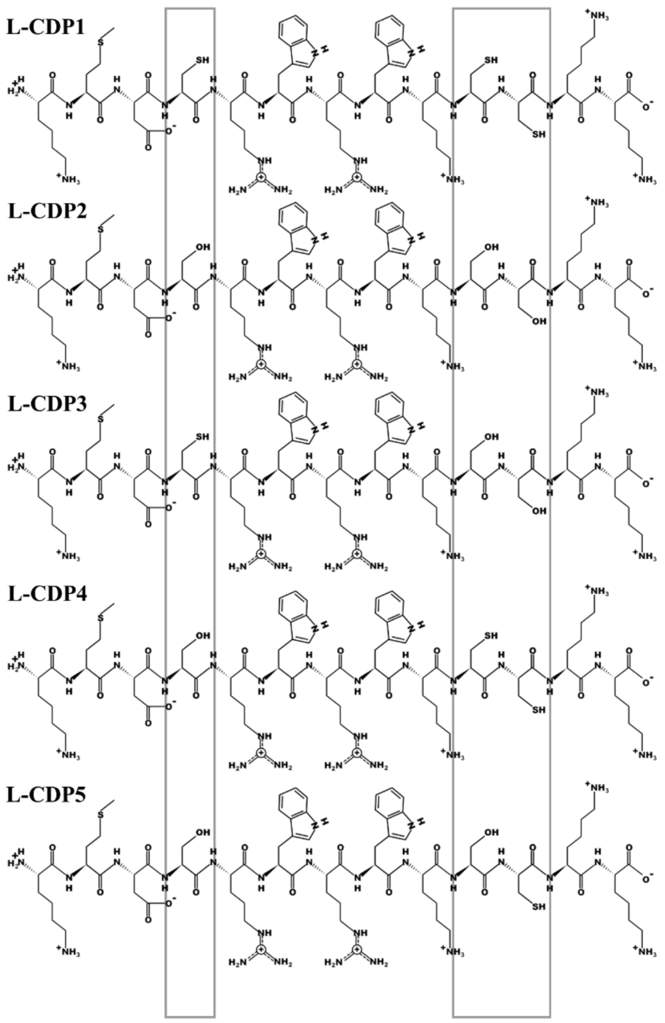

Figure S2. Primary structure of L-CDP1 to L-CDP5. Grey boxes label the amino acid variation in the sequences.

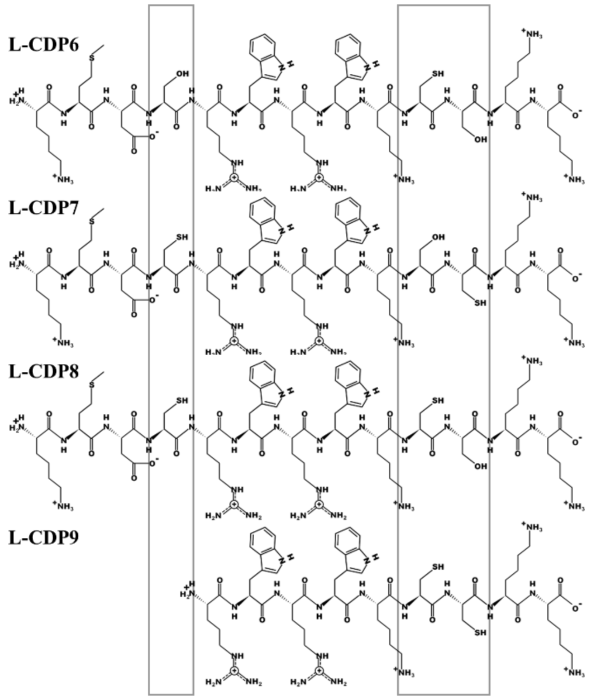

Figure S3. Primary structure of L-CDP6 to L-CDP9. Grey boxes label the amino acid variation in the sequences.

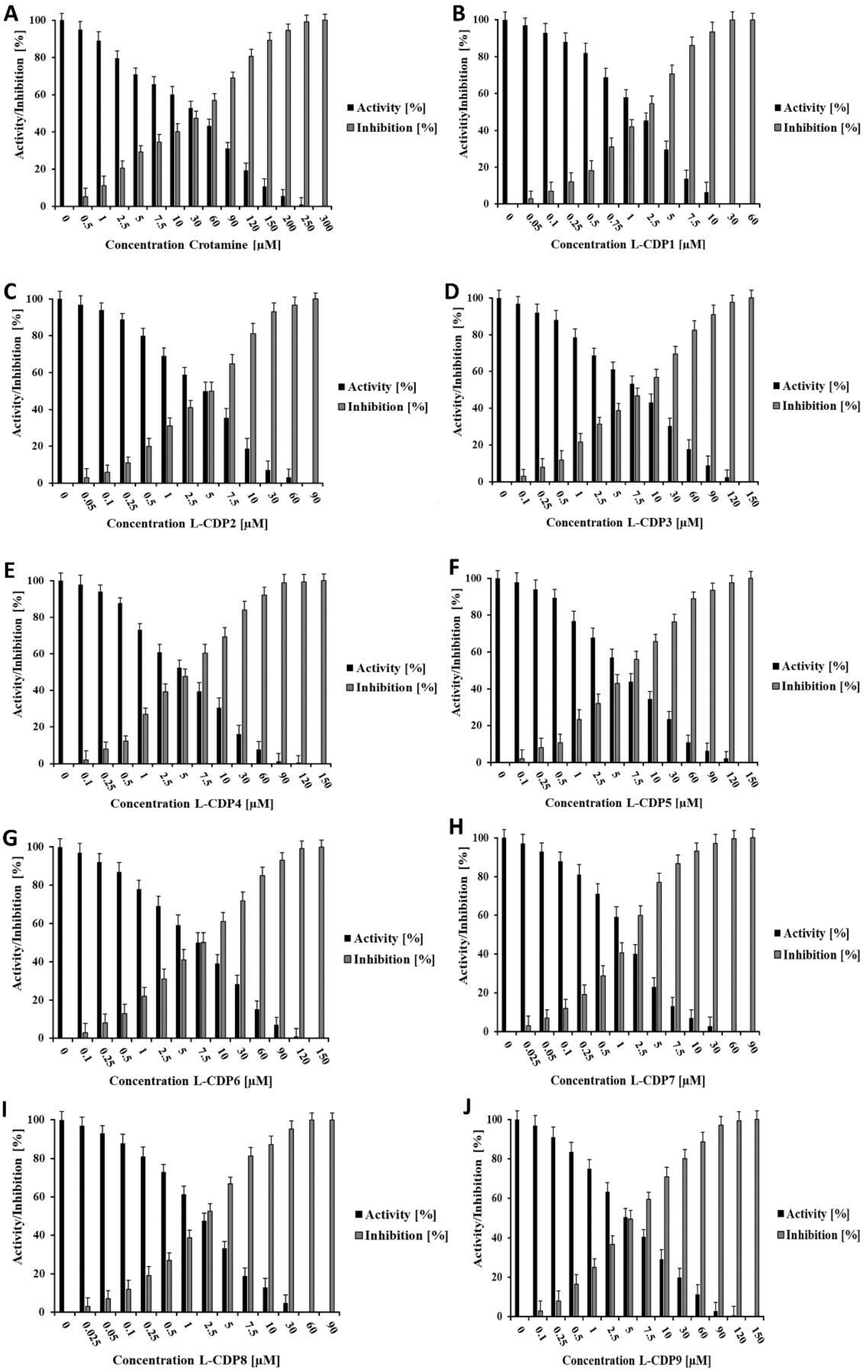

Figure S4. Inhibition effect of Crotamine and L-CDPs over SARS-CoV-2 3CL^{pro}. Normalized activity and inhibition of SARS-CoV-2 3CL^{pro} under inhibitor influence. **A:** Crotamine, **B:** L-CDP1, **C:** L-CDP2, **D:** L-CDP3, **E:** L-CDP4 and **F:** L-CDP5, **G:** L-CDP6, **H:** L-CDP7, **I:** L-CDP8 and **J:** L-CDP9. Data showed are the mean ± SD from three independent measurements (n=3).

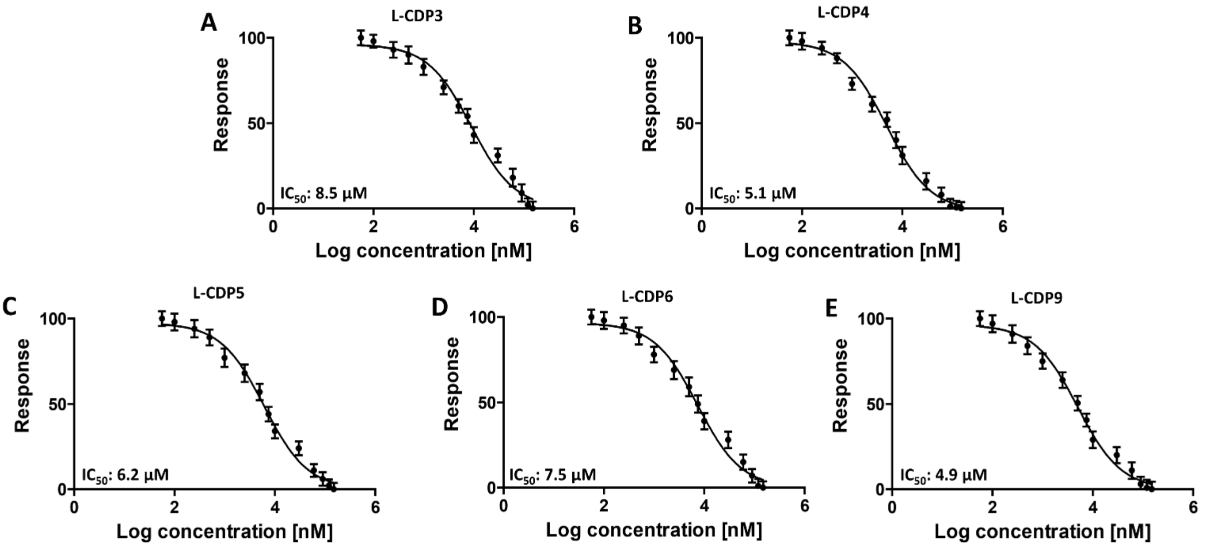

Figure S5. Crotamine and L-CDPs with inhibitory activity against SARS-CoV-2 $3CL^{pro}$. Dose-response curves for IC_{50} determination. The normalized response [%] of SARS-CoV-2 $3CL^{pro}$ is plotted against the Log of the inhibitor concentration **A:** L-CDP3, **B:** L-CDP4, **C:** L-CDP5, **D:** L-CDP6, **E:** L-CDP9. Data shown are the mean ± SD from three independent measurements (n=3).

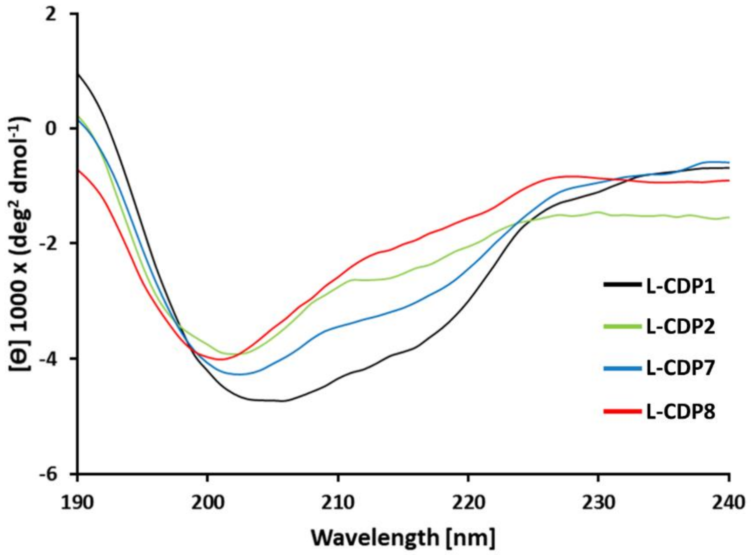

Figure S6. Circular dichroism (CD) spectroscopy of L-CDP1, L-CDP2, L-CDP7 and L-CDP8. The CD spectrum of each L-peptide in solution is presented as molar ellipticity [θ].

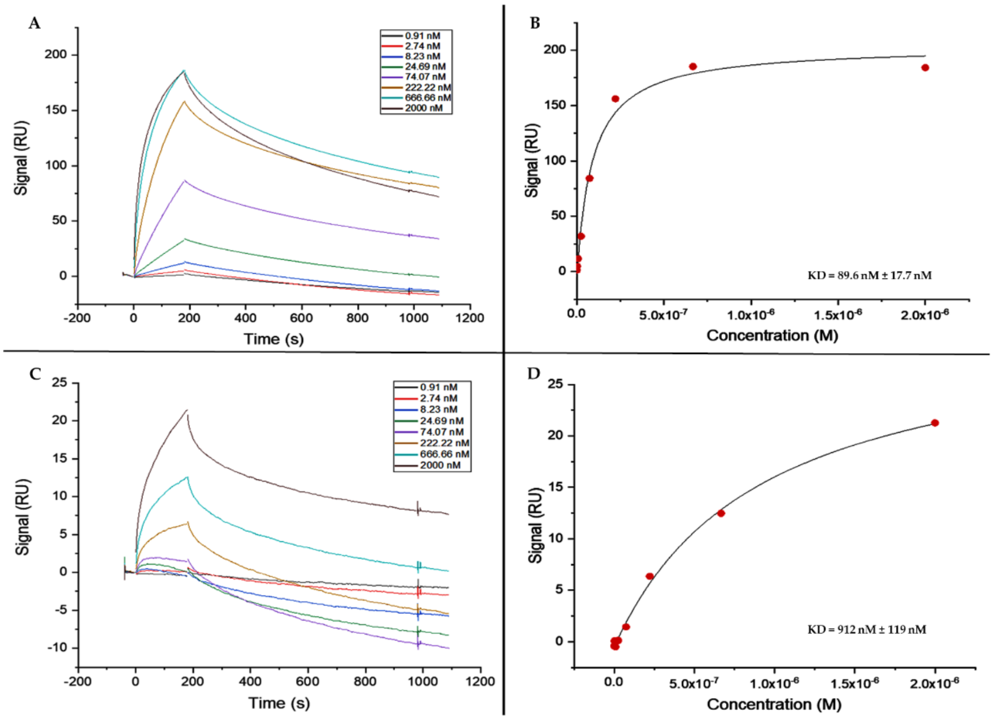

Figure S7. Dissociation constant (K_D) determination of L-CDP1 and L-CDP7 binding to SARS-CoV-2 $3CL^{pro}$ using surface plasmon resonance (SPR). **A:** SPR sensorgram of $3CL^{pro}$ and L-CDP1. **B:** Saturation curve for the L-CDP1 and $3CL^{pro}$ interaction. **C:** SPR sensorgram of $3CL^{pro}$ and L-CDP7. **D:** Saturation curve for the L-CDP7 and $3CL^{pro}$ interaction.

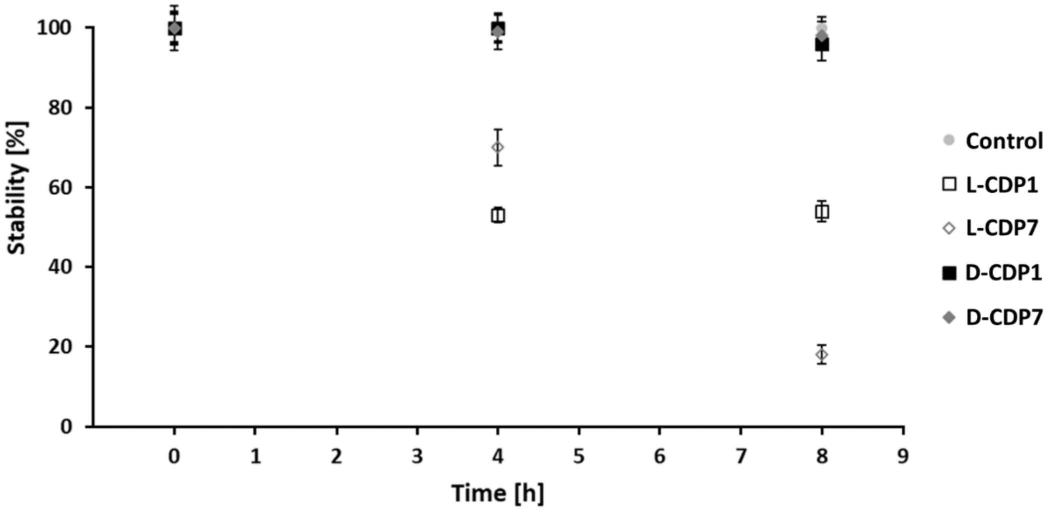

Figure S8. Stability of L- and D-CDPs against Pepsin hydrolysis. As a control, a D-peptide was used (RD2), which was described as stable against pepsin digestion [1]. Also, CDP1 and CDP7 D-peptides are stable against pepsin hydrolysis over the experimental time. In contrast, the L-CDP1 amount reduces to around 50% after 8h and L-CDP7 to around 20%.

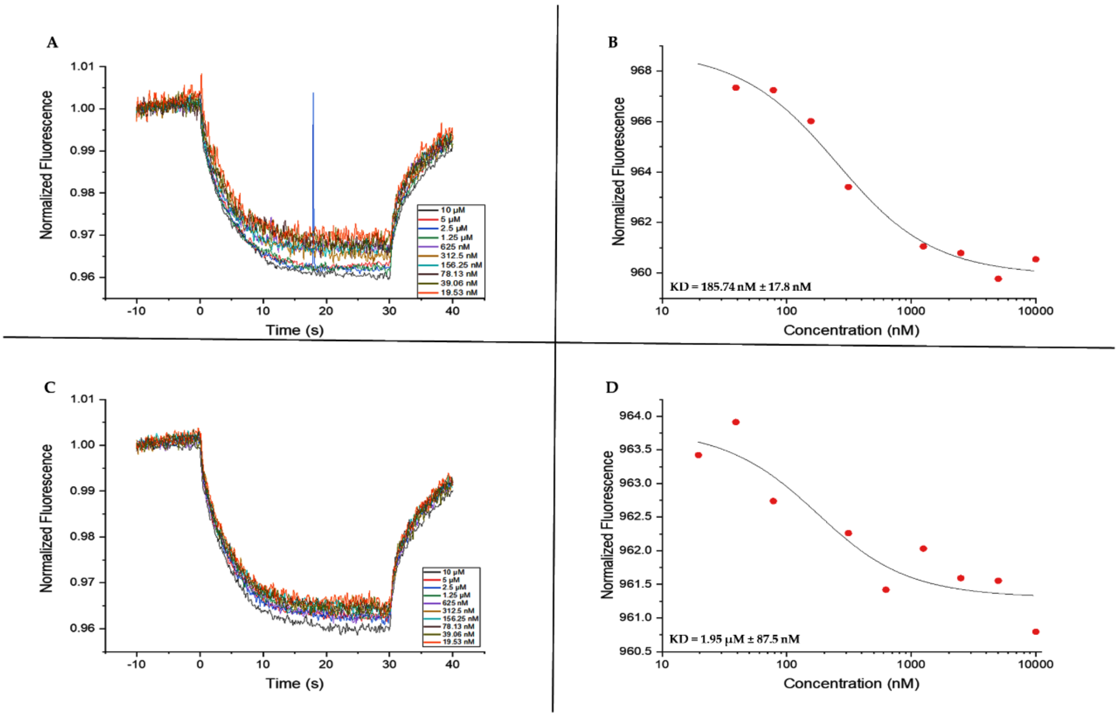

Figure S9. Dissociation constant (K_D) determination of D-CDP1 and D-CDP7 binding to SARS-CoV-2 3CL^{pro} using microscale thermophoresis (MST). **A:** Thermophoresis data from serial dilutions of D-CDP1. **B:** Binding curve of D-CDP1 with SARS-CoV-2 3CL^{pro}. **C:** Thermophoresis data from serial dilutions of D-CDP7. **D:** Binding curve of D-CDP7 with SARS-CoV-2 3CL^{pro}.

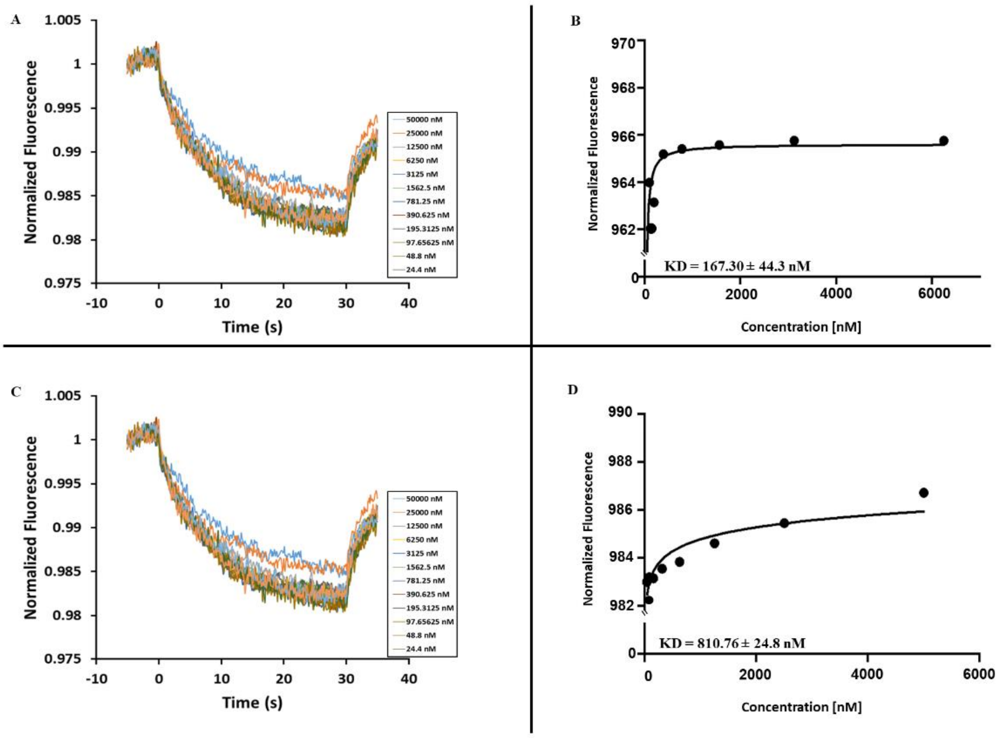

Figure S10. Dissociation constant (K_D) determination of L-CDP1 and L-CDP7 binding to SARS-CoV-2 3CL^{pro} using microscale thermophoresis (MST). **A:** Thermophoresis data from serial dilutions of L-CDP1. **B:** Binding curve of L-CDP1 with SARS-CoV-2 3CL^{pro}. **C:** Thermophoresis data from serial dilutions of L-CDP7. **D:** Binding curve of L-CDP7 with SARS-CoV-2 3CL^{pro}.

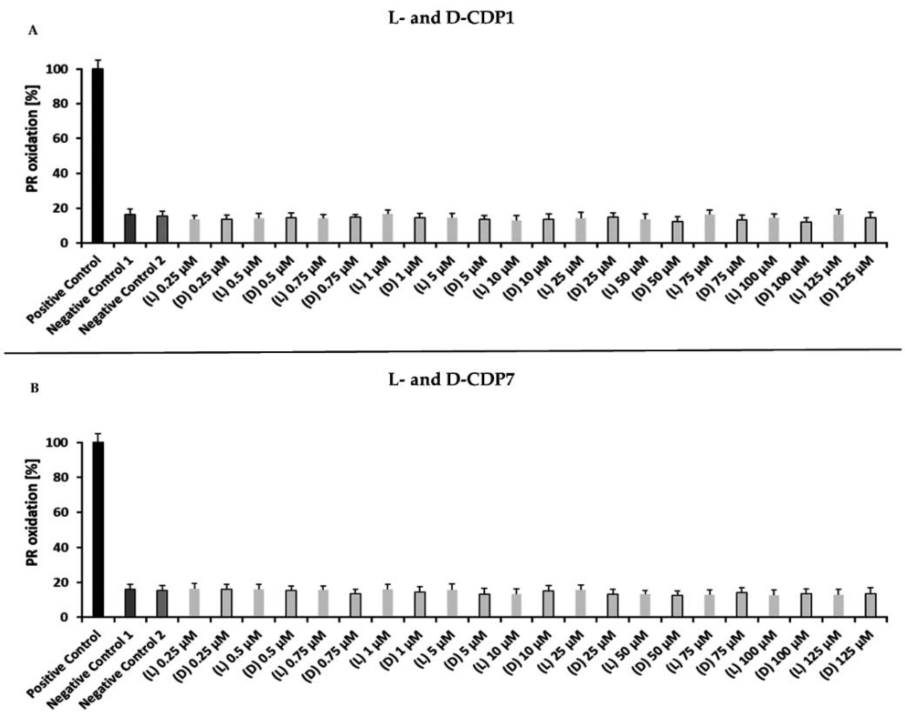

Figure S11. H_2O_2 generating capacity of L- and D-CDPs under the influence of 1 mM TCEP. The experiments were performed to measure the H_2O_2 generating capacity of L-/D-CDP1 and L-/D-CDP7 under the influence of 1mM TCEP. Further, the H_2O_2-dependent horseradish peroxidase (HRP) mediates phenol red (PR) oxidation, which can be followed at 610 nm. Positive control: HRP-PR and H_2O_2; negative control 1: HRP-PR; negative control 2: PR; Data showed are the mean ± SD from three independent measurements (n=3). **A:** H_2O_2 generation by L- and D-CDP1. **B:** H_2O_2 generation by L- and D-CDP7.

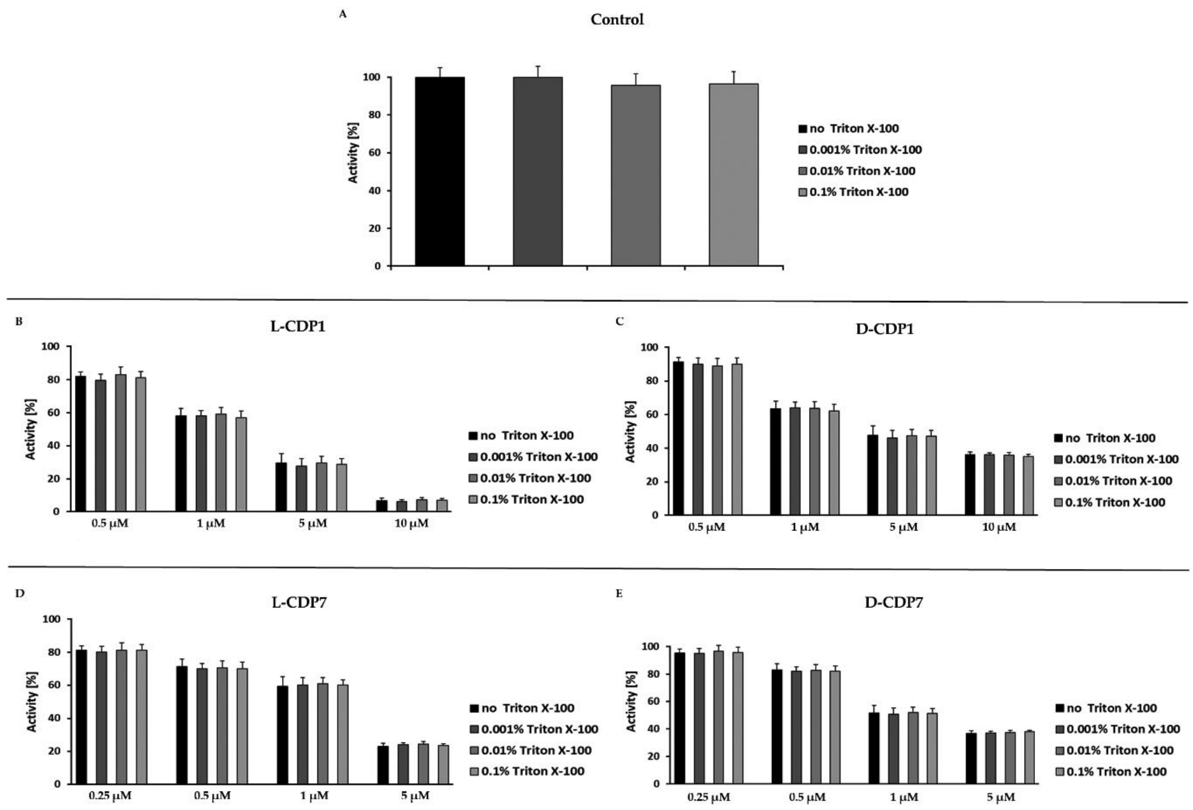

Figure S12. Effect of Triton X-100 on the L- and D-CDPs inhibition against SARS-CoV-2 3CL^{pro}. The experiments were performed to exclude the possibility that the peptides inhibit the protease promiscuously by aggregation. Three Triton X-100 concentrations (0.001%, 0.01% and 0.1%) were tested with four different inhibitor concentrations and compared without detergent. Additionally, the effect of Triton X-100 against the protease was tested. Data shown are the mean ± SD from three independent measurements (n=3). **A:** Effect of Triton X-100 on 3CL^{pro} activity. **B:** Effect of Triton X-100 on L-CDP1 inhibition against 3CL^{pro}. **C:** Effect of Triton X-100 on D-CDP1 inhibition against 3CL^{pro}. **D:** Effect of Triton X-100 on L-CDP7 inhibition against 3CL^{pro}. **E:** Effect of Triton X-100 on D-CDP7 inhibition against 3CL^{pro}.

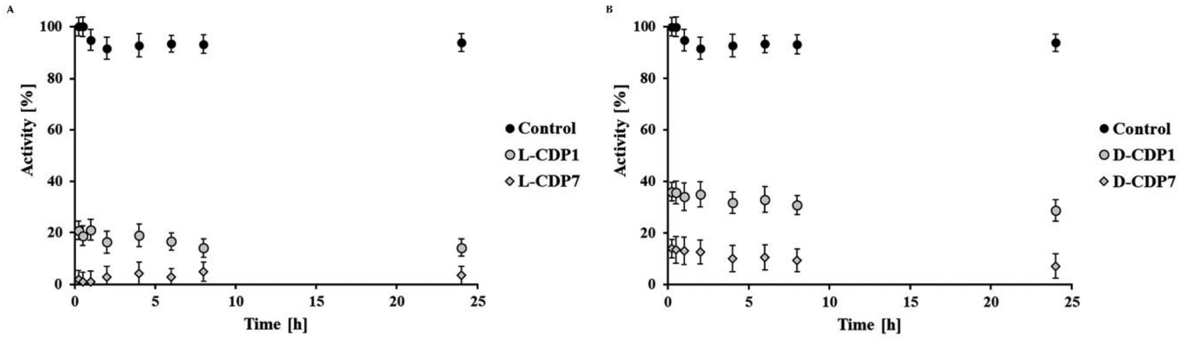

Figure S13. 24 h inhibition experiment of L- and D-CDPs against SARS-CoV-2 3CL^{pro}. **A:** 24h inhibition experiment of L-CDP1 and L-CDP7 against SARS-CoV-2 3CL^{pro}. **B:** 24h inhibition experiment of D-CDP1 and D-CDP7 against SARS-CoV-2 3CL^{pro}.

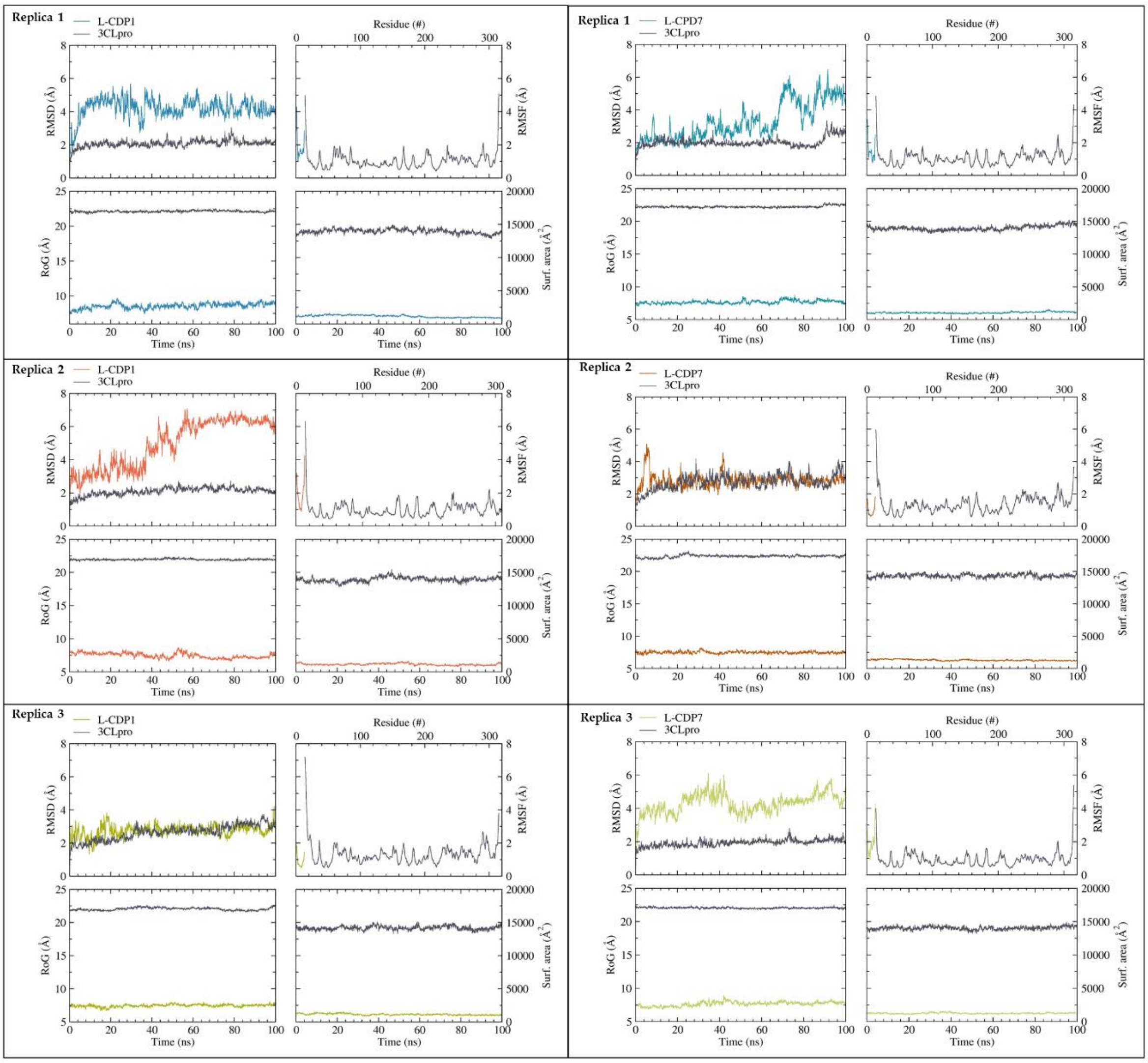

Figure S14. RMSD values and RMSF profiles for the replicate 100 ns of MD simulations of 3CL^{pro} in complex with L-CDP1 (left) and L-CDP7 (right) peptides. The RMSD time profiles concerning all backbone atoms of 3CL^{pro} and the peptides (L-CDP1 and L-CDP7). The RMSF plot corresponds to the protein/peptide complex, and the replicates are coloured differently.

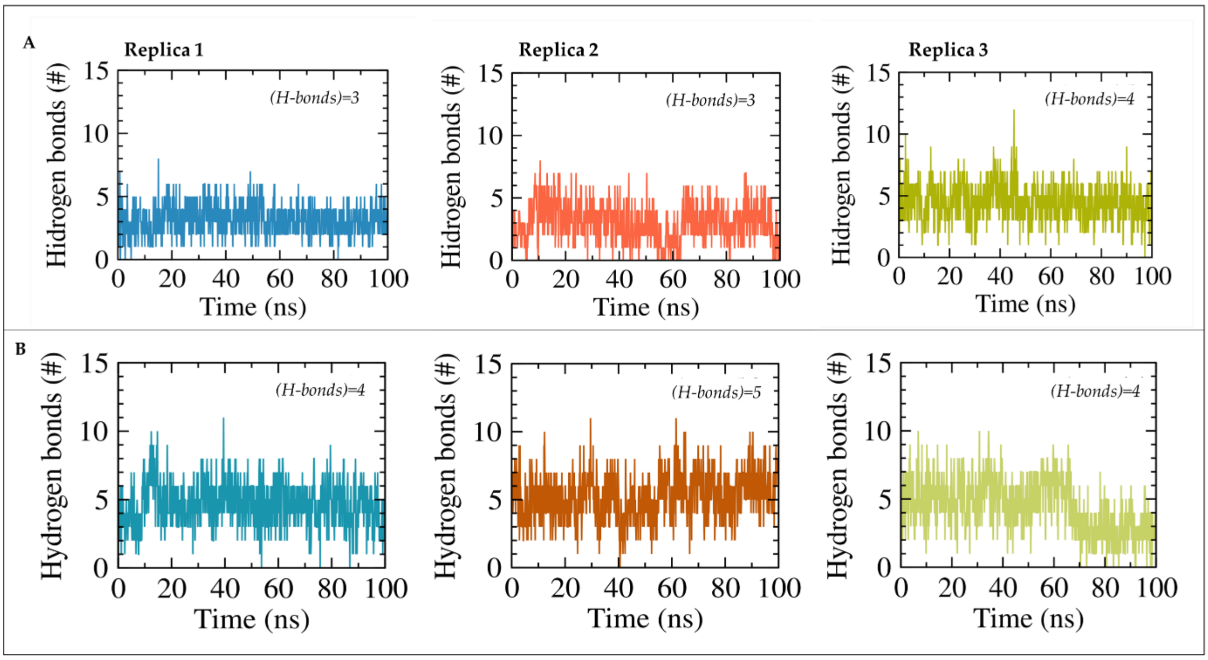

Figure S15. Intermolecular H-bonds of the replicates MD simulations: (**A**) H-bonds between the L-CDP1 peptide with 3CL^{pro} residues. (**B**) H-bonds between the L-CDP7/ 3CL^{pro} residues.

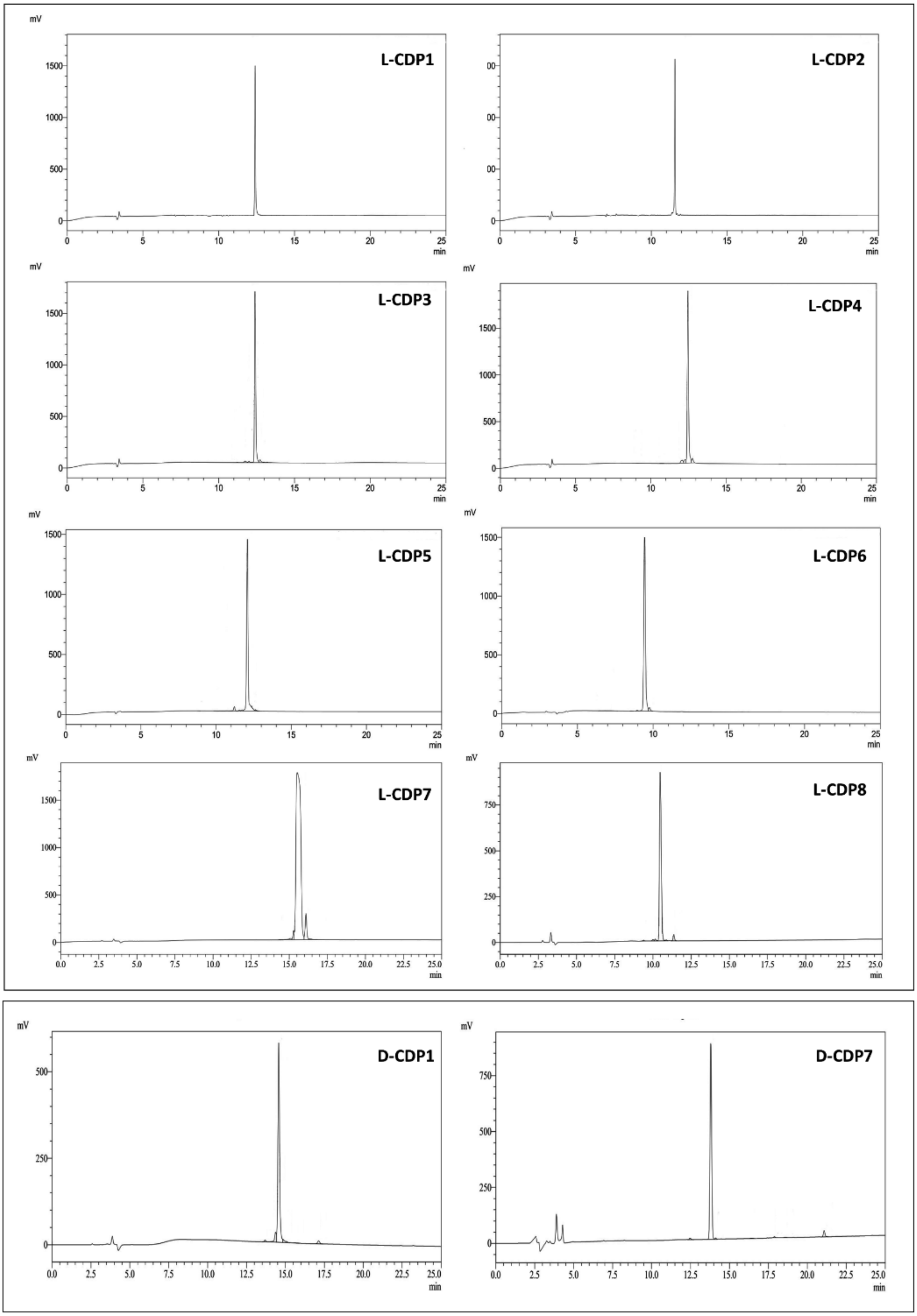

Figure S16. HPLC chromatogram of the L-CDP and D-CDP peptides.

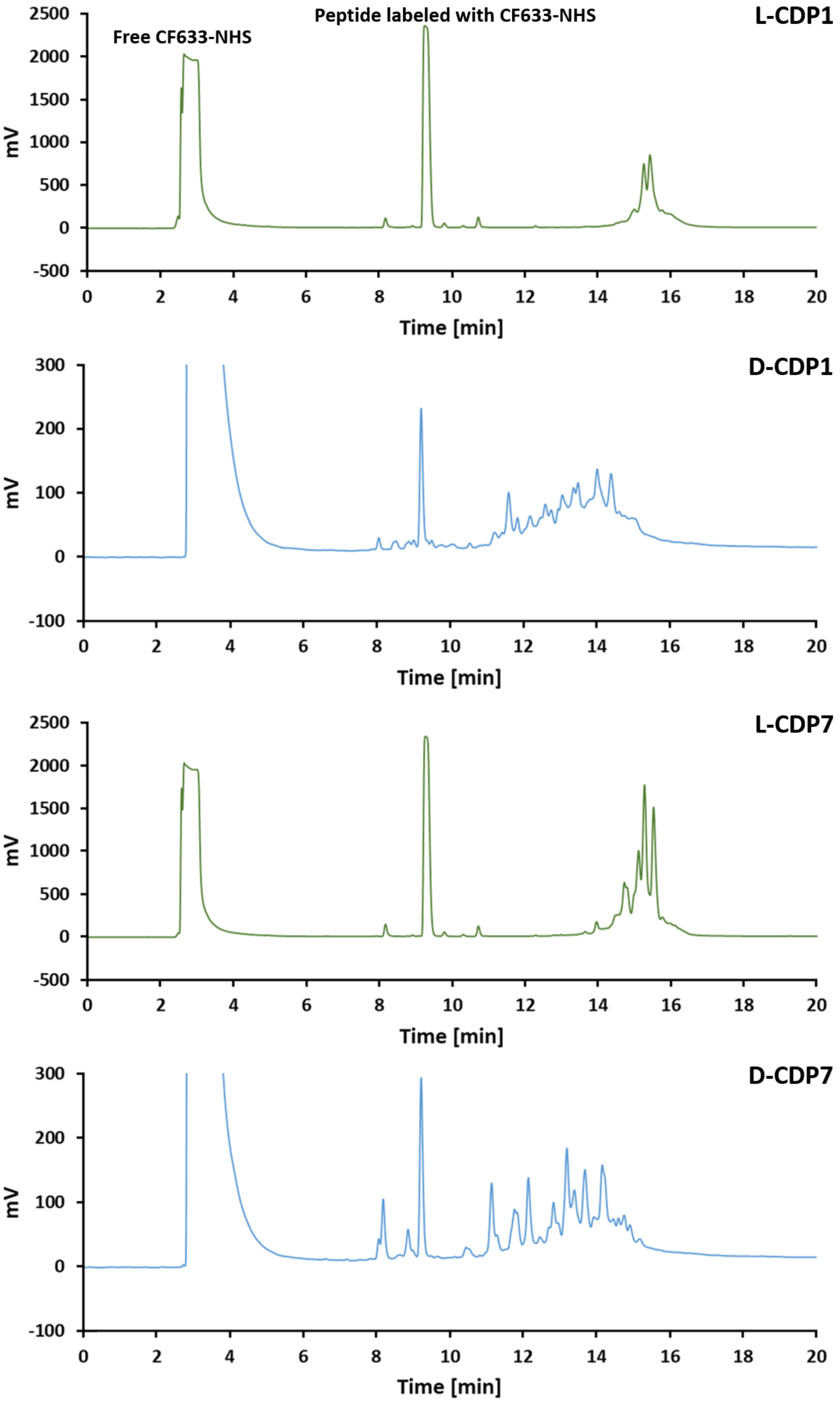

Figure S17. HPLC chromatogram of the L/D-CDP1 and L/D-CDP7 peptides after the labelling process with CF633-NHS.

Table S1. K_D determination of L-CDP1 and L-CDP7 using SPR.

Peptide	K_D1 [nM]	K_D2 [nM]	Average K_D [nM]	STD [nM]
**L-CDP1**	89.06	40.92	64.99	± 20.07
**L-CDP7**	374.3	233.7	304.0	± 70.3

Table S2. Basic information about tested L- and D-CDPs.

	Sequence	Conformation	MW	pI	Net charge	Solvent
**CDP1**	KMDCRWRWKCCKK	L and D	1769.84	10.13	+5	H_20
**CDP2**	KMDSRWRWKSSKK	L	1721.91	11.68	+5	H_20
**CDP3**	KMDCRWRWKSSKK	L	1737.89	10.99	+5	H_20
**CDP4**	KMDSRWRWKCCKK	L	1753.87	10.55	+5	H_20
**CDP5**	KMDSRWRWKSCKK	L	1737.89	10.99	+5	H_20
**CDP6**	KMDSRWRWKCSKK	L	1737.89	10.99	+5	H_20
**CDP7**	KMDCRWRWKSCKK	L and D	1753.87	10.55	+5	H_20
**CDP8**	KMDCRWRWKCSKK	L	1753.87	10.55	+5	H_20
**CDP9**	RWRWKCCKK	L	1292.67	10.83	+5	H_20

Table S3. K_D determination of D-CDP1 and D-CDP7 using MST.

Peptide	K_D1 [nM]	K_D2 [nM]	K_D3 [nM]	Average K_D [nM]	STD [nM]
**L-CDP1**	113	221.5	167.4	167.30	± 44.3
**L-CDP7**	451.3	1033	948	810.76	± 24.8
**D-CDP1**	201.09	166.24	189.88	185.74	± 17.8
**D-CDP7**	1851	2012	1991	1951.3	± 87.5

References

[1] Elfgen, A.; Hupert, M.; Bochinsky, K.; Tusche, M.; González de San Román Martin, E.; Gering, I.; Sacchi, S.; Pollegioni, L.; Huesgen, P.F.; Hartmann, R.; Santiago-Schübel, B. Metabolic resistance of the D-peptide RD2 developed for direct elimination of amyloid-β oligomers. Sci. Rep. 2019, 9, 1-13.
